# Effectiveness and cost-effectiveness of a structured social coaching intervention for people with psychosis (SCENE): protocol for a randomised controlled trial

**DOI:** 10.1136/bmjopen-2021-050627

**Published:** 2021-12-13

**Authors:** Domenico Giacco, Agnes Chevalier, Megan Patterson, Thomas Hamborg, Rianna Mortimer, Yan Feng, Martin Webber, Penny Xanthopoulou, Stefan Priebe

**Affiliations:** 1 Warwick Medical School, University of Warwick, Coventry, UK; 2 Unit for Social and Community Psychiatry, Queen Mary University of London, London, UK; 3 Pragmatic Clinical Trials Unit, Queen Mary University of London, London, UK; 4 Department of Social Policy and Social Work, University of York, York, UK; 5 Medical School, University of Exeter, Exeter, UK

**Keywords:** schizophrenia & psychotic disorders, social medicine, mental health

## Abstract

**Introduction:**

People with psychosis tend to have smaller social networks than both people in the general population and other people with long-term health conditions. Small social networks are associated with poor quality of life. Preliminary evidence suggests that coaching patients to increase their social contacts may be effective. In this study, we assessed whether structured social coaching improves the quality of life of patients with psychosis (primary outcome) compared with an active control group, receiving information on local social activities.

**Methods and analysis:**

A structured social coaching intervention was developed based on the literature and refined through stakeholder involvement. It draws on principles from motivational interviewing, solution focused therapy and structured information giving. It is provided over a 6-month period and can be delivered by a range of different mental health professionals. Its effectiveness and cost-effectiveness are assessed in a randomised controlled trial, compared with an active control group, in which participants are given an information booklet on local social activities. Participants are aged 18 or over, have a primary diagnosis of a psychotic disorder (International Classification of Disease: F20–29) and capacity to provide informed consent. Participants are assessed at baseline and at 6, 12 and 18 months after individual randomisation. The primary outcome is quality of life at 6 months (Manchester Short Assessment of Quality of Life). We hypothesise that the effects on quality of life are mediated by an increase in social contacts. Secondary outcomes are symptoms, social situation and time spent in social activities. Costs and cost-effectiveness analyses will consider service use and health-related quality of life.

**Ethics and dissemination:**

National Health Service REC London Hampstead (19/LO/0088) provided a favourable opinion. Findings will be disseminated through a website, social media, scientific papers and user-friendly reports, in collaboration with a lived experience advisory panel.

**Trial registration number:**

ISRCTN15815862.

**Strengths and limitations of this study**

- Broad inclusion criteria, allowing inclusion of patients with a range of individual characteristics (eg, varying level of financial difficulties, and different types and levels of symptoms).
- Inclusion of a large number of urban, semiurban and rural sites, as different geographical contexts may influence the availability or convenience of social activities.
- Active control condition, that is, the provision of comprehensive information on local social activities.
- During this trial, we focused on people of working age (18–65) and only included people who are fluent in English.
- Since social coaching was not part of routine care before this trial, the coaches in the experimental intervention are—although trained—not experienced in this type of approach.

## Introduction

At any given time, more than 200 000 people experience a psychotic disorder in England alone. The total costs for England was estimated to be £4 billion in 2007 and £6.5 billion by 2026.^1^ People with psychotic disorders have much smaller social network sizes compared with the general population, and compared with other groups with long-term mental and physical health problems.^2 3^ Social isolation is not only a serious problem in itself, but is also linked with poor quality of life and a range of unfavourable health outcomes.^3–6^

Traditionally, pharmacological and psychological treatments have attempted to reduce the social isolation of patients with psychosis indirectly; through treating symptoms or by teaching social skills.^7^ However, the symptoms of psychosis which are mostly linked with social isolation, that is the ‘negative symptoms’ do not show a substantial response to established pharmacological treatments.^8^ Social skills training has been found to be effective in teaching these skills to patients, however, this does not translate to improved social functioning.^9^ Given the evidence that its benefits are limited, social skills training is not recommended by National Institute for Health and Care Excellence (NICE) guidelines.^4^

A systematic review by Anderson *et al*^7^ found that interventions which directly focused on supporting socialisation activities had a positive effect on reducing social isolation.^10–13^ These interventions were diverse, including guided peer support, social coaching and dog-assisted integrative psychological therapy. The largest and highest quality trial among the ones identified tested a social coaching intervention, which was the only intervention clearly targeting social contacts outside of services.^11^ This led to the decision that this model would inform our intervention development. One of the limitations identified by the systematic review was that none of the studies reported an economic analysis of the costs and benefits of the interventions.^7^

In a research programme funded by the National Institute for Health Research (https://scene.elft.nhs.uk), we developed a manualised and structured social coaching intervention. The effective components identified in the international literature^7^ were refined and adapted to the English National Health Service (NHS) context. The intervention was designed to improve patients’ quality of life through increasing their social networks.

## Objectives

### Primary objective

To assess whether the structured social coaching intervention improves the quality of life of patients with psychosis (primary outcome) compared with an active control group, which received information on local social activities.

### Secondary objectives

1. To understand whether changes in quality of life are mediated by an increase in social contacts (in the previous week).
2. To evaluate whether the intervention improves secondary outcomes such as social contacts, symptoms, social situation, feelings of loneliness, time spent in social activities, health-related quality of life and whether it reduces service use.
3. To assess costs and cost-effectiveness of the intervention.
4. To explore implementation of the intervention.

## Methods

### Study design

Individually randomised, parallel group controlled trial. The intervention and control condition are provided in addition to standard care.

### Study sites

This multicentre study is led and sponsored by East London NHS Foundation Trust (https://www.elft.nhs.uk/Contact-Us) and includes the sites listed in box 1. The study is currently open to additional sites.

Box 1 **Trial sites**

- East London National Health Service (NHS) Foundation Trust (two sites: East London and Luton).
- Tees, Esk and Wear valleys NHS Foundation Trust.
- Devon Partnership NHS Trust.
- Cornwall NHS Partnership NHS Trust.
- Oxford Health NHS Foundation Trust.
- Somerset NHS Foundation Trust.
- Leeds and York NHS Foundation Trust.
- Humber Teaching NHS Foundation Trust.
- Gloucestershire Health and Care NHS Foundation Trust.
- Coventry and Warwickshire Partnership NHS Trust.
- Lincolnshire Partnership NHS Trust.
- Cambridgeshire and Peterborough NHS Foundation Trust
- Essex Partnership University NHS Foundation Trust

#### Eligibility criteria

##### Inclusion criteria

Patients:

- 18–65 years old.
- Diagnosis of psychosis-related condition (International Classification of Disease 10th revision, F20–29).
- Capacity to provide informed consent.
- Ability to communicate in English.
- Limited social network size (three or less social contacts with non-first degree relatives in the previous week).
- Low quality of life (score 5 or less on the Manchester Short Assessment of Quality of Life (MANSA) quality of life assessment).
- Not receiving hospital treatment at the time of recruitment.

Social coaches:

- Mental health professionals with experience of providing mental healthcare (eg, psychiatrists, clinical psychologists, nursing staff, occupational therapists), minimum NHS Band 4 or equivalent experience.
- Aged 18 and over.
- Capacity to provide informed consent.
- Ability to communicate in English.

### Intervention development

**Figure 1:**
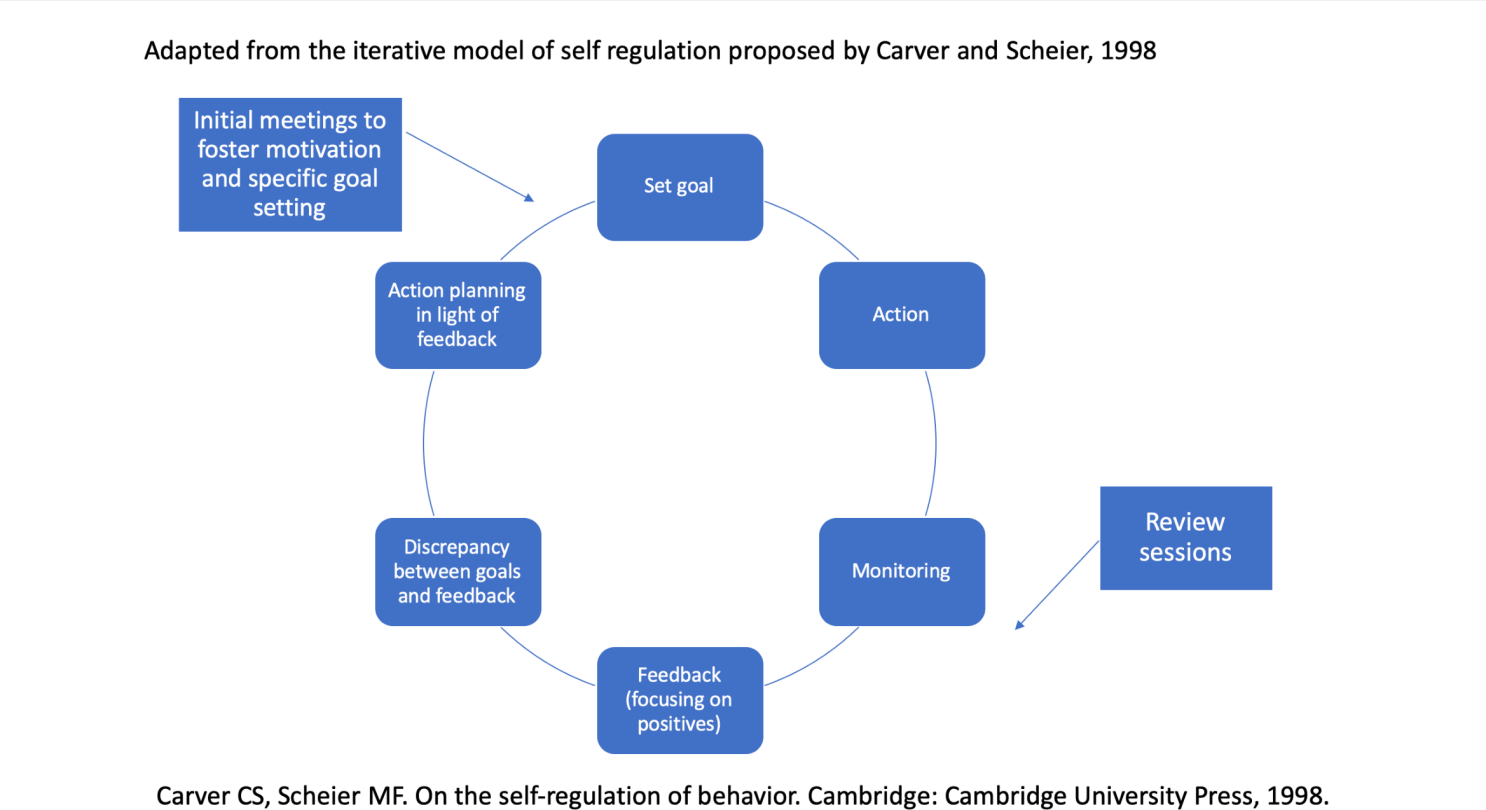
Theoretical model of intervention processes.

The intervention developed by Terzian *et al*^11^ was taken as the starting point for the intervention tested in this trial. It was specified and expanded using approaches from solution-focused therapy and motivational interviewing, and considering previous experiences of the group in developing and evaluating complex psychosocial interventions, in particular DIALOG+.^14^ It was further modified and then manualised by the Programme Management group, which included experts in psychiatry, psychology, social work, occupational therapy, social sciences and behavioural change and experts by experience. The intervention was finalised in consultation with stakeholders.^15^ The intervention is considered to be generic and not profession-specific, so that it can be delivered by different professional groups. The role of the social coach is intended to be independent of other treatment and solely focused on the task of expanding social networks. Social coaches are not meant to establish a wider or longer-term therapeutic relationship, which might interfere with other therapeutic relationships of the patients. The theoretical framework used is shown in figure 1.

### Randomisation procedures

Patients are individually randomised to either the intervention or control group. The allocation ratio is 1:1. Randomisation is stratified by site (NHS Trust), ensuring balanced numbers of patients in each group at each NHS Trust. Permuted blocked randomisation with block sizes of m=6, 4 and 2 are used within each stratum. Patients are allocated to clinicians based on locality and availability that is, not randomly. The randomisation is carried out remotely by the Pragmatic Clinical Trials Unit at Queen Mary, University of London, which is also responsible for database development and assists the team with data monitoring.

### Trial arms

#### Arm 1: intervention

##### Who delivers the intervention

The intervention is delivered by clinicians from different backgrounds (eg, psychologists/assistant psychologists, social workers, nursing staff, occupational therapists and medical doctors), with a minimum level of experience and seniority equivalent to an NHS band 4. They take up a role of ‘social coach’ for the treated patient box 2.

Box 2 **SCENE Social coaching intervention: the eight step approach**

**How are the steps covered during the sessions?**

There is some flexibility as to how much time each step takes and to what extent they are covered within the monthly sessions varies.

Session 1: Usually, the first meeting would end with steps 5 or 6.

Session 2: After the second meeting an activity is agreed upon. However, it is allowed that patients and professional leave the decision to a third session or take it during the first session if this is possible.

Session 3–6 (follow-up sessions): During follow-up sessions, social coach and patient discuss to what extent the activity has been done. The social coach provides positive feedback and—if required—deals with complete or partial perceived failure using solution focused techniques (eg, emphasising what went well).

After 3 months (ie, session 3–4), the situation is re-evaluated. If the activity is working well, then this is monitored in further follow-up sessions. In case professional and patient come to the conclusion that the originally planned activity does not work, a face-to-face meeting is arranged in which steps 4–8 of the initial meetings are repeated and a different activity is planned.

##### Type and frequency of sessions

Social coaches meet patients at least three times but ideally monthly over the 6-month intervention period. During the first two sessions of the intervention, a structured eight step approach is followed and then revisited in follow-up meetings. Please see details of the eight step approach and of the intervention sessions in box 2.

The intervention starts with two initial face-to-face sessions (which can also occur via video conferencing), each lasting between 60 and 90 min. The main aim of these initial meetings is to introduce the intervention, explore participants’ social history and discuss preferences and options for activities. The participant then selects one social activity to focus on during the remaining meetings and actions are agreed. The subsequent meetings include discussions around challenges and progress and and take place monthly, lasting about 20 min each. The final meeting is face-to-face and is used as both a summary of progress and to plan for future social activities after the intervention. The intervention can be stopped at any time on participant request.

##### Training and supervision of social coaches

Social coaches are trained in one session lasting 3 hours, normally in a group format (although one-to-one sessions can be arranged). Training is provided by senior SCENE researchers.

During the training coaches acquire knowledge of the structure and aims of the intervention, and of simple motivational interviewing (eg, identifying change talk) and solution focused therapy techniques (eg, identifying what has worked in the past). Scenarios in which barriers for the patients in engaging in new social activities may appear and strategies to overcome them are discussed.

Learning progress is assessed during the training and in the subsequent supervision, provided by senior members of the research team.

Clinicians receive updates on changes in options for activities from the local research team. They will also receive at least two supervision sessions by SCENE senior researchers.

### Arm 2: control group

Patients in the control group are provided with information about local options for social activities via a booklet sent to them in the post or handed over by a researcher. This is intended to control for the provision of information on social activities and service attention to their social isolation.

### Outcomes

#### Primary outcome

The primary outcome is subjective quality of life, measured with the MANSA at the end of the intervention period (6 months after randomisation).

The MANSA has been widely used in research and cited in more than 850 research papers. The MANSA is brief with very high completion rates and excellent psychometric properties.^16^

### Mediator of effect on primary outcome

Number of social contacts in the previous week, measured at 6 months follow-up using the Social Contacts Assessment (SCA).^17 18^

### Secondary outcomes

- Social Contacts (SCA).^17^
- Psychopathological symptoms assessed with the Positive And Negative Syndrome Scale.^19^
- Social situation with the SIX.^20^
- Feeling of loneliness with the University of California, Los Angeles (UCLA) Loneliness Scale.^21^
- Time spent in social activities with the Time Use Survey.^22^
- Health-related quality of life with the EQ-5D-5L.^23^
- Service use with the Client Service Receipt Inventory,^24^ and from NHS Digital datasets.^25^

Study outcomes and timepoints are summarised in table 1.

**Table 1:** Scene study outcomes and time points

Assessment	Screening	Baseline	Study phase (6 months)	Follow-up (12 months)	Follow-up (18 months)
All patient participants					
MANSA	x	x	x	x	x
Social contacts assessment	x	x	x	x	x
PANSS		x	x	x	x
Social situation		x	x	x	x
Loneliness		x	x	x	x
Time spent in social activities		x	x	x	x
EQ-5D-5L		x	x	x	x
Client service receipt inventory		x	x	x	x
Healthcare source use (NHS digital)		x	x	x	x
Intervention participants only					
Semistructured interviews			x		
Social coaches					
Adherence schedule			x		
Semistructured interviews			x		

MANSA, Manchester Short Assessment of Quality of Life; NHS, National Health Service; PANSS, Positive And Negative Syndrome Scale.

### Patient and public involvement

A Lived Experience Advisory Panel (LEAP) has been set up and meets every 6 months to advise on study progress, review materials and support dissemination plans.

The LEAP has a central role in the preparation of study materials, design of practical procedures and dissemination. For example, LEAP members provided valuable feedback during the development of the intervention, and for facilitating recruitment and finding out about available activities in the community. The chair of the LEAP is an expert by experience who attends regular meetings with the project team and is directly involved in parts of the research, in particular the interpretation of qualitative data. Findings from all work packages, including the intervention development are discussed with and influenced by the LEAP.

### Internal pilot

The trial comprised an internal pilot with the aim of checking the feasibility of recruiting to target. The recruitment target for the internal pilot was 140 participants representing an average rate of four participants per site per month for 5 months. This was achieved within the 5-month time frame.

### Independent committees

The trial has an independent project steering committee and a data monitoring committee. Both include among their members one clinician/clinical researcher, one quantitative methodologist and one person with lived experience of mental illness.

### Analyses

#### Statistical analysis

The primary outcome analysis will be the comparison of mean MANSA scores between treatment groups at 6 months follow-up using a heteroscedastic partially nested mixed-effects model.^26^ This model will account for clustering by treating clinician in the intervention arm, baseline values of the outcome (MANSA) and site as covariates.

Secondary outcomes will be analysed using the same model as for the primary outcome or an equivalent model appropriate for the outcome type where the secondary outcome is not continuous. Differences in outcome measures between groups will be compared for 6 months, 12 months and 18 months follow-up data. Additionally, repeated measures models comprising all four time points will be fitted. Baseline characteristics of patients will be tabulated by treatment arms.

The analysis will be on an intention-to-treat basis, and every effort will be made to collect complete data. If any outcome data are missing, available subject data only will be analysed (unbiased analysis under missing-at-random assumption); however, patterns of missing data will be explored, and a strategy for dealing with missing values will be articulated in the formal statistical analysis plan.

We are planning individual level single imputations (replacing missing values by a fixed value defined by a certain rule) analyses for partially completed primary outcome data to assess the uncertainty around the primary outcome analysis estimate. Further details of other sensitivity analyses planned will be outlined in the statistical analysis plan prior to analysis.

A mediator analysis will identify whether the effect on the primary outcome is mediated through expanded social networks (SCA) at 6 months, as hypothesised. The product of coefficients method, with a Sobel test and bootstrap standard errors will be used.^27^ Further mediation analyses will assess the mediation effect of increases in SCA at 6 months on patients’ MANSA score at 12 months follow-up.

All analyses are incorporated into a statistical analysis plan, and allocation codes will not be released to the statistician before the analysis plan is signed off. All researchers involved in developing the analysis plan will remain blinded until the analysis plan is signed off.

### Sample size calculation

It is assumed that the proposed new intervention would be implemented and funded across the NHS only if it achieved at least a medium sized effect. An effect size of 0.35 is equivalent to an improvement of satisfaction ratings on the MANSA of at least one scale point (on a 7-point scale) for 4 out of a total of 12 life domains. An improvement of quality of life in four life domains is usually regarded as a meaningful difference to patients’ life.^16^

For detecting such an effect size with 90% power, assuming a conservative intraclass correlation coefficient (ICC) of 0.07 for patients treated by the same professional in the intervention group, 229 patients in the intervention group and 229 in the control group will be required (total sample=458). A 1:1 allocation ratio has been chosen for organisational ease. This requires eight additional patients to be recruited compared with the absolute minimum required sample size with slightly uneven groups. A drop-out rate (from the study) at 6 months follow-up of 20% (in line with recent trials of similar interventions with the same patient group) (VOLUME trial) was assumed.^18^ The sample size calculation was based on 10 patients being treated and followed-up per clinician on average. Based on recruiting 12 patients per clinician the final total sample size target was 576 patients (288 per arm). Recruitment and intervention delivery to SCENE were paused during the COVID-19 pandemic making a study extension necessary (see the section Impact of the COVID-19 pandemic). For this a sample size recalculation was conducted using values observed so far for two quantities. The drop-out rate was inflated from 20% to 25% (actual rate to date 24%). Cluster size in the intervention arm was reduced from 10 to 3 patients per treating professional on average allowing for observed variability in patients per professional. Using these estimates the updated total number of patients to recruit is 504.

### Qualitative process evaluation

A qualitative process evaluation will be conducted, employing semistructured interviews with a number of purposively selected patients and social coaches. Interviews will be transcribed and analysed using thematic analysis.^28^

We will interview 40 patients in the experimental group to explore experiences of the intervention and descriptions of qualitative changes in their social network, contacts and activities. A topic guide for interviews was developed with input from the LEAP. Interviews will be conducted after the end of the 6-month outcome assessment, so that the interviews do not interfere with the effects of the intervention in influencing the primary outcome (quality of life at 6 months). Unblinded researchers will identify participants, conduct the interviews and manage qualitative data, so researchers assessing outcomes remain blind to allocation.

We will use purposive sampling, to include patients who differ according to gender, whether they live in urban or rural settings and whether or not they completed the intervention. Sampling of social coaches will include those who have seen more than three patients and those who have seen fewer.

### Adherence to manual

Adherence to manual will be assessed through our adherence checklist. Routine documentation and audiotapes of patient–professional meetings (for consenting participants) will be compared against the clinician-reported adherence schedule to check reliability. This is a self-reported checklist of whether and how the different steps of the intervention (box 2) are addressed. Clinicians will have addressed all the eight steps of the intervention and conducted at least three sessions with a given patient for the intervention to be deemed as completed.

### Economic evaluation

The within trial analysis will adopt the NHS and Personal Social Services perspective to assess the cost-effectiveness of the psychosocial intervention for patients with psychosis compared with best standard practice.^29^ The evaluation will focus on the 18 months from baseline until the end of the follow-up period. The analysis will adhere to guidelines for good economic evaluation practice as outlined in Ramsey *et al*^30^ and Sanders *et al.*^31^

Resource-use associated with delivery of the interventions in both trial arms will be identified using a specially designed intervention implementation form. Participants’ use of health services, including mental health and hospital care, will be extracted from the NHS Digital database. All participants will be asked to complete a modified version of the Client Services Receipt Inventory to obtain information about their of other psychosocial interventions, medication and receipt of informal care from families and friends. The quantities of all resource use will be combined with unit costs to generate cost at the individual participant level. Unit costs will be obtained from various sources, including a specially designed coach demographics questionnaire, NHS Reference costs,^32^ Unit Costs of Health and Social Care,^33^ NHS drug Tariff^34^ and the UK earnings data.^35^

As for outcomes, the primary outcome measure for the economic evaluation will be collected using the EQ-5D-5L instrument.^23^ We will calculate the participant-level quality-adjusted life-years (QALYs) and use the EQ-5D-5L data with the area-under-the-curve approach.^36^ The secondary outcome will be measured using the MANSA. The time points for collecting costs and outcome data are reported in table 1.

Both costs and outcomes occurring during the last 6 months of the follow-up period will be discounted at 3.5% in line with the NICE recommendation.^29^ Costs and outcome data will be analysed by treatment allocation, and differences between trial arms will be estimated over 18 months, adjusting for baseline differences using regression analysis. We will select an appropriate method to handle missing data based on the nature of our data.

Cost–utility analysis and cost-effectiveness analysis will be applied in the economic evaluation. In the cost–utility analysis, the estimates of incremental cost-effectiveness ratio (ICER) for the psychosocial intervention compared with best standard practice will be presented against the decision-maker’s willingness to pay a value of £20 000–£30 000 per QALY.^23^ To report the uncertainty of the point estimate of ICER, we will use the non-parametric bootstrap approach to estimate the CI around the ICER. We will also present the probability that the intervention is cost-effective against a range of decision makers’ willingness to pay value using the cost effectiveness acceptability curve. In the cost-effectiveness analysis, we will calculate the incremental cost per unit change on the MANSA scale and uncertainty surrounding the ratio.

A number of sensitivity analyses to assess the impact of key assumptions as well as uncertainty with key parameters in the economic evaluation will be conducted to (1) explore the impact of alternative assumptions about the missing data mechanism; (2) consider uncertainty in the most important cost drivers to assess the impact of healthcare use; (3) use a broader analytical perspective by including additional costs for informal care; (4) use 0–6 months from randomisation as the time period for economic evaluation.

### Ethics and dissemination

The study was reviewed and a favourable opinion received from the London Hampstead NHS Research Ethics Committee (19/LO/0088). Any serious adverse events are recorded in specific forms and their relationship with the intervention are adjudicated by site leads who are all senior clinicians. Written informed consent is provided by participants after discussion with researchers. A model consent form is enclosed as a online supplemental document.

10.1136/bmjopen-2021-050627.supp1 Supplementary data

Any personal information stored in locked cabinets on NHS premises if in paper version, and encrypted if in electronic version. Dataset will be accessed by the study team and after the primary analysis may be made available to other parties subject to data sharing agreements.

Throughout all phases of the research, we will disseminate information about the activities of the programme through social media and a project specific website (http://scene.elft.nhs.uk/) in order to reach a wider public audience. Authorship guidelines for outputs will follow the International Committee of Medical Journal Editors (ICMJE) guidelines.

When results become available, they will be disseminated through:

- Scientific publications in peer-reviewed open-access journals.
- Presentations at national and international conferences and to professional and non-professional audiences at appropriate events.
- Existing networks, including but not limited to the WHO, the benchmarking network in mental health, organisations involved in Quality Improvement programmes and the professional networks of the programme management group members.

### Impact of the COVID-19 pandemic

Following the outbreak of COVID-19 in the UK, recruitment was stopped from 16 March 2020 to 1 October 2020. We decided to stop the intervention delivery from 16 March 2020 to 21 August 2020 in accordance with social distancing guidelines at that time.

We added an additional follow-up at 10 months from randomisation for those participants who had already been randomised when the study was stopped, but had not completed the treatment period. A sensitivity analysis will consider end-of-treatment as the outcome of comparison, and hence include the assessment of quality of life at 10 months from randomisation rather than at 6 months for these participants.

We created additional adapted versions (used separately from the standard ones) of the SCA and of the Time Use Survey and data collected will be analysed to capture online social contacts and activities throughout the trial.

The recruitment and randomisation of participants was resumed on 1 October 2020. Additional instructions to social coaches were provided on physical distancing with patients and on how to encourage social activities which are either online or can be carried in accordance with different scenarios and different physical distancing directives.

Research follow-up of participants at different timepoints was never stopped and continued over the phone or via videoconferencing.

We have calculated that 70 participants may have been especially affected by the COVID-19 pandemic in that their primary outcome was assessed at a time when restrictions meant that they could not meet more than one person outside of their household.

To adjust for any pandemic effects on the intervention itself, the outcomes or both, a sensitivity analysis will adopt a mixed-effects model approach, grouping participants according to the physical distancing guidance that they have been exposed to. Individual treatment effect estimates of participant groups with different levels of exposure will be calculated.

## Discussion

This study addresses a gap in mental healthcare provision, that is, the lack of treatments available to help patients overcome their social isolation.^4^ It has broad inclusion criteria and is carried out across a large number of urban, semiurban and rural sites. This will allow to control by design a number of patient-level (eg, financial and clinical status) and area-level characteristics (eg, availability of affordable activities, distance and travel required to access them) which might influence intervention effectiveness. The active control condition, that is, the provision of comprehensive information on local social activities, does not only control for service attention to social activity and information provision, but also arguably represents a reflection of best current practice.

The methodological choices made when designing the study come with some limitations:

- Older people and those who are not fluent in the language of their country of residence encounter additional barriers to socialisation. Hence, we restricted inclusion criteria to patients with psychotic disorders of working age and to those who are fluent in English, to limit heterogeneity of our sample. Future trials and/or implementation studies should target these populations specifically.
- The new intervention has been implemented in services directly as part of the trial. Social coaches are trained but do not have previous experience in delivering this type of approach.

In addition to this, the COVID-19 outbreak meant that more of the intervention has to be delivered remotely than originally envisaged, and we do not know whether and how that will impact on outcomes. We have a sensitivity analysis to estimate this as explained in detail in the following paragraph.

If the intervention is found to be effective in increasing social contacts and improving quality of life, it can promptly become part of the therapeutic armamentarium of mental health services. The flexibility of the approach, which can be delivered by different type of professionals, might facilitate its uptake within the ever evolving landscape of mental health services. In line with the design of the trial, we do not intend for the social coach to become be a new professional role in itself. Instead, the function of social coaches can be taken on by different professionals and exercised along with other clinical activities.

## Supplementary Material

Author's manuscript
