# Supplementary material for: Effectiveness and cost-effectiveness of a structured social coaching intervention for people with psychosis (SCENE): protocol for a randomised controlled trial
Source: BMJ Open. 2021 Dec 13;11(12):e050627. doi: 10.1136/bmjopen-2021-050627 (PMC8671980; doi:10.1136/bmjopen-2021-050627)
Supplement: Supplementary data [file bmjopen-2021-050627supp001.pdf]

SCENE
Enhancing social networks, improving quality of life

[INSERT TRUST LOGO]

**Service User Consent Form**
**Randomised Control Trial of a Structured Intervention For Expanding Social Networks in Psychosis (SCENE WP5)**

Please **initial** box (OR researcher to tick for remote consent)

1. I confirm that I have read and understand the information sheet dated 24/09/20 Version 3.0 for the above study. I have had the opportunity to ask the researcher questions and these questions have been answered to my satisfaction. ☐

2. I understand that my participation is voluntary and that I am free to withdraw at any time without giving any reason and without my treatment or legal rights being affected. ☐

3. I agree that if I withdraw, or am withdrawn from the study for any reason, then researchers can continue to use the information I have already given them unless I contact them before the end of July 2022 ☐

4. I understand that if I decide to stop the SCENE intervention, researchers can contact me to find out the reasons why but I am under no obligation to share this information. ☐

5. I understand that all information will be kept confidential. My personal data will only be accessed by the study team on a need to know basis. The research data will be kept for 20 years but from this, I can only be identified by a study ID code. I understand that confidentiality will need to be broken if there is a concern for risk to other people or to myself, or if criminal disclosures are made. ☐

6. I agree that members of the research team at East London NHS Foundation Trust may access my medical records to obtain basic information about me. ☐

7. I agree to take part in the above study. ☐

Please **turn over** to finish completing this form

SCENE WP5 – Service User Consent Form v.2.0 24.09.20
IRAS ID: 257667

Items 8-11 are **optional**. You do not have to agree to these if you do not want to. Please only **initial the boxes** for the items that you agree to.

8. I agree to be interviewed about my experiences of the SCENE intervention and for my interview to be audio recorded. I understand that the recording will be typed-up and any personal information destroyed. I agree that anonymised quotations from the interviews will be used to share the research findings. ☐

9. I agree for initial sessions of the SCENE intervention with a trained mental health professional to be audio-recorded. I understand that I will be asked for permission before each recorded session and that I am free to refuse or stop the recording at any time. ☐

10. I understand that personal information collected about me including date of birth, NHS number and postcode will be sent to the sponsor to obtain information about my service use for the purpose of this research. ☐

11. I agree to my General Practitioner (GP) being informed of my participation in this study. ☐

| | | |
|---|---|---|
| Name of Participant | Date | Signature<br>(OR *provided electronically by the researcher, on behalf of the participant for remote consent)* |

I have explained the study to the participant and have answered the participant's questions honestly and fully

| | | |
|---|---|---|
| Name of Researcher | Date | Signature |

Original for investigator site file, 1 copy for participant, 1 copy for medical records

SCENE WP5 – Service User Consent Form v.2.0 24.09.20
IRAS ID: 257667
