# Supplementary material for: Effectiveness and cost-effectiveness of a structured social coaching intervention for people with psychosis (SCENE): protocol for a randomised controlled trial
Source: BMJ Open. 2021 Dec 13;11(12):e050627. doi: 10.1136/bmjopen-2021-050627 (PMC8671980; doi:10.1136/bmjopen-2021-050627)
Supplement: Author's manuscript [file bmjopen-2021-050627.draft_revisions.pdf]

BMJ Open is committed to open peer review. As part of this commitment we make the peer review history of every article we publish publicly available.

When an article is published we post the peer reviewers' comments and the authors' responses online. We also post the versions of the paper that were used during peer review. These are the versions that the peer review comments apply to.

The versions of the paper that follow are the versions that were submitted during the peer review process. They are not the versions of record or the final published versions. They should not be cited or distributed as the published version of this manuscript.

BMJ Open is an open access journal and the full, final, typeset and author-corrected version of record of the manuscript is available on our site with no access controls, subscription charges or pay-per-view fees (http://bmjopen.bmj.com).

If you have any questions on BMJ Open's open peer review process please email info.bmjopen@bmj.com

Effectiveness and cost-effectiveness of a structured social coaching intervention for people with psychosis (SCENE): trial protocol

Journal:	*BMJ Open*
Manuscript ID	bmjopen-2021-050627
Article Type:	Protocol
Date Submitted by the Author:	26-Feb-2021
Complete List of Authors:	Giacco, Domenico; University of Warwick; Newham Centre for Mental Health, Unit for Social and Community Psychiatry<br>Chevalier, Agnes; Newham Centre for Mental Health, Unit for Social and Community Psychiatry<br>Patterson, Megan; Newham Centre for Mental Health, Unit for Social and Community Psychiatry<br>Hamborg, Thomas; Queen Mary University of London<br>Mortimer, Rianna; Queen Mary University of London<br>Feng, Yan; Queen Mary University of London, Blizard Institute<br>Webber, Martin; University of York, Department of Social Policy and Social Work<br>Xanthopoulou, Penny; University of Exeter, Medical School<br>Priebe, Stefan; Barts and the London School of Medicine and Dentistry, University of London, Unit of Social and Community Psychiatry
Keywords:	Schizophrenia & psychotic disorders < PSYCHIATRY, SOCIAL MEDICINE, MENTAL HEALTH

SCHOLARONE™
Manuscripts

I, the Submitting Author has the right to grant and does grant on behalf of all authors of the Work (as defined in the below author licence), an exclusive licence and/or a non-exclusive licence for contributions from authors who are: i) UK Crown employees; ii) where BMJ has agreed a CC-BY licence shall apply, and/or iii) in accordance with the terms applicable for US Federal Government officers or employees acting as part of their official duties; on a worldwide, perpetual, irrevocable, royalty-free basis to BMJ Publishing Group Ltd ("BMJ") its licensees and where the relevant Journal is co-owned by BMJ to the co-owners of the Journal, to publish the Work in this journal and any other BMJ products and to exploit all rights, as set out in our licence.

The Submitting Author accepts and understands that any supply made under these terms is made by BMJ to the Submitting Author unless you are acting as an employee on behalf of your employer or a postgraduate student of an affiliated institution which is paying any applicable article publishing charge ("APC") for Open Access articles. Where the Submitting Author wishes to make the Work available on an Open Access basis (and intends to pay the relevant APC), the terms of reuse of such Open Access shall be governed by a Creative Commons licence – details of these licences and which Creative Commons licence will apply to this Work are set out in our licence referred to above.

Other than as permitted in any relevant BMJ Author's Self Archiving Policies, I confirm this Work has not been accepted for publication elsewhere, is not being considered for publication elsewhere and does not duplicate material already published. I confirm all authors consent to publication of this Work and authorise the granting of this licence.

For peer review only

Effectiveness and cost-effectiveness of a structured social coaching intervention for people with psychosis (SCENE): trial protocol

3912/4000 words

Authors:

Domenico Giacco,[1,2] Agnes Chevalier,[2] Megan Patterson,[2] Thomas Hamborg,[3] Rianna Mortimer,[3] Yan Feng,[3] Martin Webber,[4] Penny Xanthopoulou,[5] Stefan Priebe.[2]

Affiliations:

[1] Division of Health Sciences, Warwick Medical School, University of Warwick, Coventry, CV4 7AL

[2] Unit for Social and Community Psychiatry, (WHO Collaborating Centre for Mental Health Service Development), Barts and the London School of Medicine, Queen Mary University of London, Newham Centre for Mental Health, London, E13 8SP

[3] Institute of Population Health Sciences, Barts and the London School of Medicine, Queen Mary University of London, Yvonne Carter Building, 58 Turner Street, London E1 2AB, UK.

[4] International Centre for Mental Health Social Research, Department of Social Policy and Social Work, University of York, Heslington, York, YO10 5DD, UK.

[5] College of Medicine and Health, University of Exeter. St Luke's Campus, Exeter, EX2 4TH, UK.

Correspondence to Dr Domenico Giacco; domenico.giacco@warwick.ac.uk

For peer review only

ABSTRACT (294 words)

Introduction

People with psychosis tend to have smaller social networks than both people in the general population and other people with long-term health conditions. Small social networks are associated with poor quality of life. At present no interventions are directly aimed at, or effective in, increasing patients' social networks, and through that improving their quality of life and other health outcomes. Preliminary evidence suggests that coaching patients to increase their social contacts may be effective.

Methods and analysis

A structured social coaching intervention was developed based on the literature and refined through stakeholder involvement. It draws on principles from motivational interviewing, solution focused therapy and structured information-giving. It is provided over a six month period and can be delivered by a range of different mental health professionals. Its effectiveness and cost-effectiveness are assessed in a randomised controlled trial, compared to an active control group, in which participants are given an information booklet on local social activities.

Participants are aged 18 or over, have a primary diagnosis of a psychotic disorder (International Classification of Disease: F20-29) and capacity to provide informed consent. Participants are assessed at baseline and at six, 12 and 18 months after individual randomisation. The primary outcome is quality of life at six months (Manchester Short Assessment of Quality of Life). We hypothesise that the effects on quality of life are mediated by an increase in social contacts. Secondary outcomes are symptoms, social situation and time spent in social activities. Costs and cost-effectiveness analyses will consider service use and health-related quality of life.

Ethics and dissemination

NHS REC London Hampstead [19/LO/0088] provided a favourable opinion. Findings will be disseminated through a website, social media, scientific papers and user-friendly reports, in collaboration with a lived experience advisory panel.

Registration

ISRCTN registry (15815862), https://doi.org/10.1186/ISRCTN15815862

For peer review only

SUMMARY

Strengths

- This study assesses a novel intervention, directly aimed at reducing social isolation to improve quality of life. No evidence-based interventions are currently available to reduce social isolation among people with severe mental illness.
- There is an active control condition, i.e. the provision of comprehensive information on local social activities.
- The trial has broad inclusion criteria and is carried out across a large number of urban, semi-urban and rural sites. This will allow us to test the intervention taking into account: a) A wide range of individual patient characteristics which may influence the effectiveness of the intervention (financial difficulties, types and levels of symptoms). b) Different geographical contexts which may influence the availability or convenience of social activities (e.g. limited availability of affordable social activities and/or long distances to cover in order to access activities in some areas).

Limitations

- During this trial we focused on people of working age (18-65) and only included people who are fluent in English. Older people and those who are not fluent in the language of their country of residence encounter additional barriers to socialisation which would have made the population too heterogeneous. Future studies should target these populations specifically.
- Since social coaching was not part of routine care before this trial, the coaches in the experimental intervention are – although trained – not experienced in this type of approach.

For peer review only

INTRODUCTION

At any given time, more than 200,000 people experience a psychotic disorder in England alone.[1] People with psychotic disorders have much smaller social network sizes compared to the general population, and compared to other groups with long-term mental and physical health problems.[2-3] Social isolation is not only a serious problem in itself, but is also linked with poor quality of life and a range of unfavourable health oucomes.[3-6]

Traditionally, pharmacological and psychological treatments have attempted to reduce the social isolation of patients with psychosis indirectly; through treating symptoms or by teaching social skills.[7] However, the symptoms of psychosis which are mostly linked with social isolation, i.e. the "negative symptoms" do not show a substantial response to established pharmacological treatments.[8] Social skills training has been found to be effective in teaching these skills to patients, however this does not translate to improved social functioning.[9] Given the evidence that itsbenefits are limited, social skills training is not recommended by NICE guidelines.[4]

A systematic review by Anderson et al.[7] found that interventions which directly focused on supporting socialisation activities had a positive effect on reducing social isolation.[10-13] These interventions were diverse, including guided peer support, social coaching, and dog-assisted integrative psychological therapy. The largest and highest quality trial among the ones identified tested a social coaching intervention.[11] In this study, a mental health professional, supported the patient to identify areas of interest for social activities outside of mental health services and provided coaching sessions as the patient engaged in them.

In a research programme funded by the National Institute for Health Research (NIHR) (https://scene.elft.nhs.uk), we developed a manualised and structured social coaching intervention. The effective components identified in the international literature[7] were refined and adapted to the English NHS context. The intervention was designed to improve patients' quality of life through increasing their social networks.

OBJECTIVES

Primary objective

To assess whether the structured social coaching intervention improves the quality of life of patients with psychosis (primary outcome) compared to an active control group, which received information on local social activities.

Secondary objectives

1. To understand whether changes in quality of life are mediated by an increase in social contacts (in the previous week).

For peer review only

2. To evaluate whether the intervention improves secondary outcomes such as social contacts, symptoms, social situation, feelings of loneliness, time spent in social activities, health-related quality of life and whether it reduces service use.
3. To assess costs and cost-effectiveness of the intervention.
4. To explore implementation of the intervention.

METHODS

Study design

Individually randomised, parallel group controlled trial. The intervention and control condition are provided in addition to standard care.

Study sites

This multi-centre study is led by East London NHS Foundation Trust and includes the sites listed in Table 1. The study is currently open to additional sites.

[Insert Table 1 here]

Eligibility criteria

Inclusion criteria

Patients:

- 18-65 years old
- Diagnosis of psychosis-related condition (ICD-10 F20-29)
- Capacity to provide informed consent
- Ability to communicate in English
- Limited social network size (three or less social contacts with non-first degree relatives in the previous week)
- Low quality of life (Score 5 or less on the MANSA quality of life assessment)
- Not receiving hospital treatment at the time of recruitment

Social coaches:

- Mental health professionals with experience of providing mental health care (e.g. psychiatrists, clinical psychologists, nursing staff, occupational therapists), minimum NHS Band 4 or equivalent experience.
- Aged 18 and over
- Capacity to provide informed consent
- Ability to communicate in English

Intervention development

The intervention developed by Terzian et al.[11] was taken as the starting point for the intervention tested in this trial. It was specified and expanded using approaches from solution-

For peer review only

focused therapy and motivational interviewing, and considering previous experiences of the group in developing and evaluating complex psycho-social interventions, in particular DIALOG+.[14] It was further modified and then manualised by the Programme Management group, which included experts in psychiatry, psychology, social work, occupational therapy, social sciences and behavioural change and experts by experience. The intervention was finalised in consultation with stakeholders.[15] The intervention is considered to be generic and not profession-specific, so that it can be delivered by different professional groups. The role of the social coach is intended to be independent of other treatment and solely focused on the task of expanding social networks. Social coaches are not meant to establish a wider or longer-term therapeutic relationship, which might interfere with other therapeutic relationships of the patients. Intervention procedures and components are reported in Box 1.

Randomisation procedures

Patients are individually randomised to either the intervention or control group. The allocation ratio is 1:1. Randomisation is stratified by site (NHS Trust), ensuring balanced numbers of patients in each group at each NHS Trust. Permuted blocked randomisation with block sizes of m=6, 4 and 2 are used within each stratum. Patients are allocated to clinicians based on locality and availability i.e. not randomly.The randomisation is carried out remotely by the Pragmatic Clinical Trials Unit at Queen Mary, University of London.

Trial arms

<u>Arm 1. Intervention</u>

a. *Who delivers the intervention*

The intervention is delivered by clinicians from different backgrounds (e.g. psychologists/assistant psychologists, social workers, nursing staff, occupational therapists and medical doctors), with a minimum level of experience and seniority equivalent to a NHS band 4. They take up a role of “social coach” for the treated patient.

b. *Type and frequency of sessions*

Social coaches meet patients at least three times but ideally monthly over the six-month intervention period. During the first two sessions of the intervention, a structured eight step approach is followed and then revisited in follow-up meetings. Please see details of the eight step approach in Box 1.

[Insert box 1 here]

The intervention starts with two initial face-to-face sessions (which can also occur via video conferencing), each lasting between 60 and 90 minutes. The main aim of these initial meetings is to introduce the intervention, explore participants’ social history and discuss preferences and options for activities. The participant then selects one social activity to focus on during the remaining meetings and actions are agreed. The subsequent meetings include discussions around challenges and progress and and take place monthly, lasting about 20 minutes each.

For peer review only

The final meeting is face-to-face and is used as both a summary of progress and to plan for future social activities after the intervention.

c. *Training and supervision of social coaches*

Social coaches are trained in one session lasting three hours, normally in a group format (although one-to-one sessions can be arranged). Training is provided by senior SCENE researchers.

During the training coaches acquire knowledge of the structure and aims of the intervention, and of simple motivational interviewing (e.g. identifying change talk) and solution focused therapy techniques (e.g. identifying what has worked in the past). Scenarios in which barriers for the patients in engaging in new social activities may appear and strategies to overcome them are discussed.

Learning progress is assessed during the training and in the subsequent supervision, provided by senior members of the research team.

Clinicians receive updates on changes in options for activities from the local research team. They will also receive at least two supervision sessions by SCENE senior researchers.

<u>Arm 2. Control group</u>

Patients in the control group are provided with information about local options for social activities via a booklet sent to them via post or handed over by a researcher. This is intended to control for the provision of information on social activities and service attention to their social isolation.

Outcomes

<u>Primary Outcome</u>

The primary outcome is subjective quality of life, measured with the Manchester Short Assessment of Quality of Life (MANSA) at the end of the intervention period (6 months after randomisation).

The MANSA has been widely used in research and cited in more than 850 research papers. The MANSA is brief with very high completion rates and excellent psychometric properties.[16]

<u>Mediator of effect on primary outcome</u>

Number of social contacts in the previous week, measured at 6 months follow-up using the Social Contacts Assessment (SCA).[17-18]

<u>Secondary outcomes</u>

- Social Contacts (SCA).[17]

For peer review only

- Psychopathological symptoms assessed with the Positive And Negative Syndrome Scale (PANSS).[19]
- Social situation with the SIX.[20]
- Feeling of loneliness with the UCLA Loneliness Scale.[21]
- Time spent in social activities with the Time Use Survey.[22]
- Service use with the Client Service Receipt Inventory,[23] and from NHS Digital datasets.[24]

Study outcomes and timepoints are summarised in Table 2.

[Insert Table 2 here]

Patient and public involvement

A Lived Experience Advisory Panel (LEAP) has been set-up and meets every six months to advise on study progress, review materials and support dissemination plans.

The LEAP has a central role in the preparation of study materials, design of practical procedures, and dissemination. For example, LEAP members provided valuable feedback during the development of the intervention, and for facilitating recruitment and finding out about available activities in the community. The chair of the LEAP is an expert by experience who attends regular meetings with the project team and is directly involved in parts of the research, in particular the interpretation of qualitative data. Findings from all work packages, including the intervention development are discussed with and influenced by the LEAP.

Internal pilot

The trial comprised an internal pilot with the aim of checking the feasibility of recruiting to target. The recruitment target for the internal pilot was 140 participants representing an average rate of four participants per site per month for five months. This was achieved within the 5 month time frame.

Trial registration

The trial was registered with the ISRCTN (15815862). Whilst listed restrospectively, the initial request for registration was made on the 27/02/2019, ahead of the recruitment start date and thus considered to have been prospectively registered according to ICMJE guidelines.

ANALYSES

Statistical Analysis

The primary outcome analysis will be the comparison of mean MANSA scores between treatment groups at 6 months follow-up using a heteroscedastic partially nested mixed-effects model.[25] This model will account for clustering by treating clinician in the intervention arm, baseline values of the outcome (MANSA) and *site* as covariates.

For peer review only

Secondary outcomes will be analysed using the same model as for the primary outcome or an equivalent model appropriate for the outcome type where the secondary outcome is not continuous. Differences in outcome measures between groups will be compared for 6 month, 12 month and 18 month follow-up data. Additionally, repeated measures models comprising all four time points will be fitted. Baseline characteristics of patients will be tabulated by treatment arms.

The analysis will be on an intention-to-treat basis, and every effort will be made to collect complete data. If any outcome data are missing, available subject data only will be analysed (unbiased analysis under missing-at-random assumption); however, patterns of missing data will be explored, and a strategy for dealing with missing values will be articulated in the formal statistical analysis plan.

We are planning individual level single imputations (replacing missing values by a fixed value defined by a certain rule) analyses for partially completed primary outcome data to assess the uncertainty around the primary outcome analysis estimate. Further details of other sensitivity analyses planned will be outlined in the statistical analysis plan prior to analysis.

A mediator analysis will identify whether the effect on the primary outcome is mediated through expanded social networks (SCA) at six months, as hypothesised. The product of coefficients method, with a Sobel test and bootstrap standard errors will be used.[26] Further mediation analyses will assess the mediation effect of increases in SCA at 6 months on patients' MANSA score at 12 month follow-up.

All analyses are incorporated into a statistical analysis plan, and allocation codes will not be released to the statistician before the analysis plan is signed off. All researchers involved in developing the analysis plan will remain blinded until the analysis plan is signed off.

Sample size calculation

It is assumed that the proposed new intervention would be implemented and funded across the NHS only if it achieved at least a medium sized effect. An effect size of 0.35 is equivalent to an improvement of satisfaction ratings on the MANSA of at least one scale point (on a 7 point scale) for 4 out of a total of 12 life domains. An improvement of quality of life in 4 life domains is usually regarded as a meaningful difference to patients' life.[16]
For detecting such an effect size with 90% power, assuming a conservative ICC of 0.07 for patients treated by the same professional in the intervention group, 229 patients in the intervention group and 229 in the control group will be required (total sample = 458). This sample size has been calculated using an iterative search algorithm. Initially the required sample size for the pre-specified clinically relevant improvement and power for a range of different pre-specified allocation ratios is calculated and the sample size in the intervention arm then inflated to account for the clustering due to participants being treated by the same clinician. Following this,the minimal sample size resulting in equal group sizes is identified. This requires 8 additional patients to be recruited compared to the absolute minimum required (with slightly uneven groups). We will be assuming a drop-out rate (from the study) at 6 months follow-up of 20% (in line with recent trials of similar interventions with the same patient group) (VOLUME trial).[18] The sample size calculation is based on 10 patients being treated and

For peer review only

followed-up per clinician on average. To account for drop-out, 12 patients need to be allocated to each clinician and therefore at least 24 social coaches need to be recruited to participate in the study. Based on recruiting 12 patients per clinician the final total sample size is 576 (288 per arm).

Qualitative process evaluation

A qualitative process evaluation will be conducted, employing semi-structured interviews with a number of purposively selected patients and social coaches. Interviews will be transcribed and analysed using thematic analysis.[27]

We will interview 40 patients in the experimental group to explore experiences of the intervention and descriptions of qualitative changes in their social network, contacts and activities. A topic guide for interviews was developed with input from the LEAP. Interviews will be conducted after the end of the six-month outcome assessment, so that the interviews do not interfere with the effects of the intervention in influencing the primary outcome (quality of life at six months). Un-blinded researchers will identify particpants, conduct the interviews and manage qualitative data, so researchers assessing outcomes remain blind to allocation
We will use purposive sampling, to include patients who differ according to gender, whether they live in urban or rural settings andwhether or not they completed the intervention. Sampling of social coaches will include those who have seen more than three patients and those who have seen fewer.

Adherence to manual

Adherence to manual will be assessed through our adherence checklist. Routine documentation and audiotapes of patient-professional meetings (for consenting participants) will be compared against the clinician-reported adherence schedule to check reliability. This is a self-reported checklist of whether and how the different steps of the intervention (Box 1) are addressed. Clinicians will have addressed all the eight steps of the intervention and conducted at least three sessions with a given patient for the intervention to be deemed as completed.

Economic evaluation

The within trial analysis will adopt the NHS and Personal Social Services perspective to assess the cost-effectiveness of the psychosocial intervention for patients with psychosis compared to best standard practice.[28] The evaluation will focus on the 18 months from baseline until the end of the follow-up period. The analysis will adhere to guidelines for good economic evaluation practice as outlined in Ramsey et al.[29] and Sanders et al.[30]

Resource-use associated with delivery of the interventions in both trial arms will be identified using a specially designed intervention implementation form. Participants' use of health services, including mental health and hospital care, will be extracted from the NHS Digital database. All participants will be asked to complete a modified version of the Client Services Receipt Inventory to obtain information about their of other psychosocial interventions,

For peer review only

medication and receipt of informal care from families and friends. The quantities of all resource use will be combined with unit costs to generate cost at the individual participant level. Unit costs will be obtained from various sources, including a specially designed coach demographics questionnaire, NHS Reference costs,[31] Unit Costs of Health and Social Care,[32] NHS drug Tariff,[33] and the UK earnings data.[34]

As for outcomes, the primary outcome measure for the economic evaluation will be collected using the EQ-5D-5L instrument.[35] We will calculate the participant level Quality Adjusted Life Years (QALYs) and use the EQ-5D-5L data with the area-under-the-curve approach.[36] The secondary outcome will be measured using the MANSA. The time points for collecting costs and outcome data are reported in Table 2.

Both costs and outcomes occurring during the last 6 months of the follow-up period will be discounted at 3.5% in line with the NICE recommendation.[28] Costs and outcome data will be analysed by treatment allocation, and differences between trial arms will be estimated over 18-months, adjusting for baseline differences using regression analysis. We will select an appropriate method to handle missing data based on the nature of our data.

Cost-utility analysis and cost-effectiveness analysis will be applied in the economic evaluation. In the cost-utility analysis, the estimates of incremental cost-effectiveness ratio (ICER) for the psychosocial intervention compared to best standard practice will be presented against the decision maker's willingness to pay a value of £20,000 to £30,000 per QALY.[23] To report the uncertainty of the point estimate of ICER, we will use the non-parametric bootstrap approach to estimate the confidence interval around the ICER. We will also present the probability that the intervention is cost-effective against a range of decision makers' willingness to pay value using the cost effectiveness acceptability curve. In the cost-effectiveness analysis, we will calculate the incremental cost per unit change on the MANSA scale and uncertainty surrounding the ratio.

A number of sensitivity analyses to assess the impact of key assumptions as well as uncertainty with key parameters in the economic evaluation will be conducted to (1) explore the impact of alternative assumptions about the missing data mechanism; (2) consider uncertainty in the most important cost drivers to assess the impact of healthcare use; (3) use a broader analytical perspective by including additional costs for informal care; (4) use 0 to 6 months from randomisation as the time period for economic evaluation.

ETHICS AND DISSEMINATION

The study was reviewed and a favourable opinion received from the London Hampstead NHS Research Ethics Committee [19/LO/0088]. Throughout all phases of the research, we will disseminate information about the activities of the programme through social media and a project specific website (http://scene.elft.nhs.uk/) in order to reach a wider public audience.

When results become available, they will be disseminated through:

- scientific publications in peer-reviewed open-access journals;
- presentations at national and international conferences and to professional and non-professional audiences at appropriate events;

For peer review only

- existing networks, including but not limited to the World Health Organisation, the benchmarking network in mental health, organisations involved in QI programme and the professional networks of the programme management group members.

IMPACT OF THE COVID PANDEMIC

Following the outbreak of COVID-19 in the United Kingdom, recruitment was stopped from 16/3/2020 to 01/10/2020. We decided to stop the intervention delivery from 16/3/2020 to 21/8/2020 in accordance with social distancing guidelines at that time.

We added an additional follow-up at 10 months from randomisation for those participants who had already been randomised when the study was stopped, but had not completed the treatment period. A sensitivity analysis will consider end-of-treatment as the outcome of comparison, and hence include the assessment of quality of life at 10 months from randomisation rather than at 6 months for these participants.

We created additional adapted versions (used separately from the standard ones) of the Social Contact Assessment and of the Time Use Survey and data collected will be analysed to capture online social contacts and activities throughout the trial.

The recruitment and randomisation of participants was resumed on 01/10/2020. Additional instructions to social coaches were provided on physical distancing with patients and on how to encourage social activities which are either online or can be carried in accordance with different scenarios and different physical distancing directives.

Research follow-up of participants at different timepoints was never stopped and continued over the phone or via video-conferencing.

To adjust for any pandemic effects on the intervention itself, the outcomes or both, a sensitivity analysis will adopt a mixed-effects model approach, grouping participants according to the physical distancing guidance that they have been exposed to. Individual treatment effect estimates of participant groups with different levels of exposure will be calculated.

DISCUSSION

This study addresses a gap in mental health care provision, i.e. the lack of treatments available to help patients overcome their social isolation[4]. It has broad inclusion criteria and is carried out across a large number of urban, semi-urban and rural sites. This will allow to control by design a number of patient-level (e.g. financial and clinical status) and area-level characteristics (e.g. availability of affordable activities, distance and travel required to access them) which might influence intervention effectiveness. The active control condition, i.e. the provision of comprehensive information on local social activities, does not only control for service attention to social activity and information provision, but also arguably represents a reflection of best current practice.

The methodological choices made when designing the study come with some limitations:

- Older people and those who are not fluent in the language of their country of residence encounter additional barriers to socialisation. Hence, we restricted inclusion criteria to patients with psychotic disorders of working age and to those who are fluent in English,

For peer review only

to limit heterogeneity of our sample. Future trials and/or implementation studies should target these populations specifically.
- The new intervention has been implemented in services directly as part of the trial. Social coaches are trained but do not have previous experience in delivering this type of approach.

In addition to this, the COVID-19 outbreak meant that more of the intervention has to be delivered remotely than originally envisaged, and we do not know whether and how that will impact on outcomes. We have a sensitivity analysis to estimate this as explained in detail in the following paragraph.

If the intervention is found to be effective in increasing social contacts and improving quality of life, it can promptly become part of the therapeutic armamentarium of mental health services. The flexibility of the approach, which can be delivered by different type of professionals, might facilitate its uptake within the ever evolving landscape of mental health services. In line with the design of the trial, we do not intend for the social coach to become be a new professional rolein itself. Instead, the function of social coaches can be taken on by different professionals and exercised along with other clinical activities.

AUTHORS' CONTRIBUTIONS

Giacco led the study protocol development and is the Chief Investigator. Priebe led the intervention development and is the programme co-lead. Mortimer and Hamborg led the statistical analysis plans. Feng led the health economy analysis section. All authors provided intellectual input to the paper and approved its final version.

FUNDING STATEMENT

This research is funded by the National Institute for Health Research Programme Grants for Applied Research funding stream (award number: RP-PG-0615-20009). This article reflects views of the authors and not necessarily those of the NIHR.

COMPETING INTERESTS STATEMENT

The authors declare no competing interests.

REFERENCES

1 McCrone P, Dhanasiri S, Patel A, et al. Paying the Price: The Cost of Mental Health Care in England to 2026. King's Fund 2008. https://www.kingsfund.org.uk/sites/default/files/Paying-the-Price-the-cost-of-mental-health-care-England-2026-McCrone-Dhanasiri-Patel-Knapp-Lawton-Smith-Kings-Fund-May-2008_0.pdf [accessed Feb 2021]

2 Palumbo C, Volpe U, Matanov A, et al. Social: networks of patients with psychosis: a systematic review. *BMC Res Notes* 2015;8:560.

3 Degnan A, Berry K, Sweet D, et al. Social networks and symptomatic and functional outcomes in schizophrenia: a systematic review and meta-analysis. *Soc Psychiatry Psychiatr Epidemiol* 2018; 53(9):873-888.
4 National Institute for Health and Care Excellence (NICE). Psychosis and Schizophrenia in Adults: prevention and management 2014; [CG178]. https://www.nice.org.uk/guidance/cg178/evidence/full-guideline-490503565 [accessed Feb 2021]
5 Bengtsson-Tops A, Hansson L. Quantitative and qualitative aspects of the social network in schizophrenic patients living in the community. Relationship to sociodemographic characteristics and clinical factors and subjective quality of life. *International Journal of Social Psychiatry* 2001;47(3):67-77.
6 Norman R.M, Malla A.K, Manchanda R, et al. Social support and three-year symptom and admission outcomes for first episode psychosis. *Schizophrenia Research* 2005; 80(2-3):227-34.
7 Anderson K, Laxhman N, Priebe S. Can mental health interventions change social networks? A systematic review. *BMC Psychiatry (Open Access)* 2015; 15:297.
8 Fusar-Poli P, Papanastasiou E, Stahl D, et al. Treatments of Negative Symptoms in Schizophrenia: Meta-Analysis of 168 Randomized Placebo-Controlled Trials. *Schizophr Bull* 2015; 41(4):892-9.
9 Almerie M.Q, Okba Al Marhi M, Jawoosh M, et al. Social skills programmes for schizophrenia. *Cochrane Database of Systematic Reviews* 2015; 2015(6):CD009006
10 Castelein S, Bruggeman R, Van Busschbach JT, et al. The effectiveness of peer support groups in psychosis: A randomized controlled trial. *Acta Psychiatr Scand* 2008;118:64–72
11 Terzian E, Tognoni G, Bracco R, et al. Social Network Intervention in Patients With Schizophrenia and Marked Social Withdrawal: A Randomized Controlled Study. *Can J Psychiatry* 2013;58:622–631.
12 Sheridan AJ, Drennan J, Coughlan B et al., Improving social functioning and reducing social isolation and loneliness among people with enduring mental illness: Report of a randomised controlled trial of supported socialisation. *Int J Soc Psychiatry* 2014;61(3):241-50.
13 Hasson-Ohayon I, Mashiach-Eizenberg M, Avidan M, et al. Social Cognition and Interaction Training: Preliminary Results of an RCT in a Community Setting in Israel. *Psychiatr Serv* 2014;65:555–558.
14 Giacco D, Priebe S. Using Routine Quality of Life Assessment to Improve Effectiveness of Community Mental Health Care. In: Awad AG, Voruganti LNP, eds. Beyond Assessment of Quality of Life in Schizophrenia. Springer International Publishing Switzerland 2016:145-155.
15 Tee H, Priebe S, Santos C, et al. Helping people with psychosis to expand their social networks: the stakeholders' views. *BMC Psychiatry* 2020;20(1):29.
16 Priebe S, Kellye L, Omer S, et al. The Effectiveness of a Patient-Centred Assessment with a Solution-Focused Approach (DIALOG+) for Patients with Psychosis: A Pragmatic Cluster-Randomised Controlled Trial in Community Care. *Psychotherapy and Psychosomatics* 2015;84:304-313.

For peer review only

17 Giacco D, Palumbo C, Strappelli N, et al. Social contacts and loneliness in people with psychotic and mood disorders. *Compr Psychiatry* 2016;66:59-66.
18 Priebe S, Chevalier A, Hamborg T, et al. Effectiveness of a volunteer befriending programme for patients with schizophrenia: randomised controlled trial. The British Journal of Psychiatry. Cambridge University Press; 2020;217(3):477–83.
19 Kay S. Positive and Negative Syndromes in Schizophrenia. New York: Brunner/Mazel 1991.
20 Priebe S, Watzke S, Hansson L, et al. Objective social outcomes index (SIX): a method to summarise objective indicators of social outcomes in mental health care. *Acta Psychiatr Scand* 2008;118(1):57-63.
21 Hays R.D, DiMatteo M.R. A short-form measure of loneliness. *Journal of personality assessment* 1987;51(1):69-81.
22 Priebe S, Pavlickova H, Eldridge S, et al. Effectiveness of one-to-one volunteer support for patients with psychosis: protocol of a randomised controlled trial. *BMJ open* 2016;6(8).
23 Beecham J, Knapp M. Costing psychiatric interventions. In: Thornicroft G, eds. Measuring Mental Health Needs. Gaskell, 2nd edition 2001:200-224.
24 NHS Digital. Secondary Uses Service (SUS). Available from https://digital.nhs.uk/services/secondary-uses-service-sus [accessed Feb 2021].
25 Candlish J, Teare M.D, Dimairo M, et al. Appropriate statistical methods for analysing partially nested randomised controlled trials with continuous outcomes: a simulation study. *BMC Med Res Methodol* 2018;18:105.
26 Fritz CO, Morris PE, Richler JJ. Effect size estimates: current use, calculations, and interpretation. *J Exp Psychol Gen* 2012;141(1):2-18.
27 Braun V, Clarke V. Using thematic analysis in psychology. *Qualitative Research in Psychology* 2006;3(2):77–101.
28 National Institute for Health and Care Excellence (NICE). Guide to the methods of technology appraisal 2013; https://www.nice.org.uk/process/pmg9/resources/guide-to-the-methods-of-technology-appraisal-2013-pdf-2007975843781 [accessed Aug 2020]
29 Ramsey S.D, Willke R.J, Glick H, et al. Cost-effectiveness analysis alongside clinical trials II-an ISPOR good research practices task force report. *Value Health* 2015;18(2):161–172.
30 Sanders GD, Neumann PJ, Basu A, et al. Recommendations for conduct, methodological practices, and reporting of cost-effectiveness analyses: second panel on cost-effectiveness in health and medicine. JAMA 2016;316(10):1093–1103.
31 NHS improvement. National Cost Collection for the NHS. https://www.england.nhs.uk/national-cost-collection/ [accessed Aug 2020]
32 Curtis L, Burns A. Unit Costs of Health and Social Care 2019; https://www.pssru.ac.uk/project-pages/unit-costs/unit-costs-2019/ [accessed Aug 2020]
33 NHS Business Service Authority. Drug Tariff online. https://www.nhsbsa.nhs.uk/pharmacies-gp-practices-and-appliance-contractors/drug-tariff [accessed Aug 2020]

For peer review only

34 Office for National Statistics. Earnings and working hours. https://www.ons.gov.uk/employmentandlabourmarket/peopleinwork/earningsandworkinghours [accessed Aug 2020]
35 Herdman M, Gudex C, Lloyd A, et al. Development and preliminary testing of the new five-level version of EQ-5D (EQ-5D-5L) *Qual Life Res* 2011;20:1727–36.
36 Matthews JN, Altman DG, Campbell MJ, et al. Analysis of serial measurements in medical research. *BMJ* 1990;300:230–235

Table 1. Trial sites

East London NHS Foundation Trust (two sites: East London and Luton)
Tees, Esk and Wear valleys NHS Foundation Trust
Devon Partnership NHS Trust
Cornwall NHS Partnership NHS Trust
Oxford Health NHS Foundation Trust
Somerset NHS Foundation Trust
Leeds and York NHS Foundation Trust
Humber Taching NHS Foundation Trust
Gloucestershire Health and Care NHS Foundation Trust

For peer review only

Table 2. SCENE Study outcomes and timepoints.

Assessment	Screening	Baseline	Study phase (6 months)	Follow up (12 months)	Follow up (18 months)
All Patient Participants					
MANSA	x	x	x	x	x
Social Contacts Assessment	x	x	x	x	x
PANSS		x	x	x	x
Social situation		x	x	x	x
Loneliness		x	x	x	x
Time spent in social activities		x	x	x	x
EQ-5D-5L		x	x	x	x
Client Service Receipt Inventory		x	x	x	x
Healthcare source use (NHS Digital)		x	x	x	x
Intervention Participants only					
Semi-structured interviews			x		
Social coaches					
Adherence schedule			x		
Semi-Structured Interviews			x		

For peer review only

Box 1. SCENE Social coaching intervention: the eight step approach

1) Introduction: The social coach and the patient introduce themselves.

2) Clarification of the remit of the intervention: The professional explains and discusses the focused remit of the intervention, i.e. that it aims to expand social networks and that all other therapeutic issues have to be addressed elsewhere.

3) Exploration of past and current activities: The social coach explores past activities that involved social contacts; this should be done chronologically covering the adult life time of the patient from the age of 15 years onwards, and stepwise for periods of 5 – 10 years. At the end of the exploration, professional and patient go through the list of activities (if any) and discuss to what extent the patient enjoyed each activity. Solution focused therapy techniques (e.g. identifying what went well and wat worked) can be used.

4) Motivation for change: The professional explores and discusses the patient's motivation to change and expand their social networks. Motivational interviewing techniques (e.g. identifying change talk) can be used.

5) Options for activities: Professional and patient discuss which new activities (or expanding existing ones respectively) the patient considers.

6) Information: The professional provides as much helpful information as possible about options in the given locality for patients' preferred activities. Professional and patient discuss the practicalities and sometimes decide to obtain further information. In this step, the patient is encouraged and supported to find information him/herself. Yet, if this is a substantial hurdle, the professional provides as much direct support as needed.

7) Consideration and decision: Once options have been identified, the patient is asked to consider taking it up. If the patient is ambivalent, patients are encouraged to take time, e.g. a week until the next meeting or a phone call, to think about it.

8) Definition of activity: Finally, the patient decides on the type of activity and some specification of the actual steps (e.g. twice per week attending a certain class, but not necessarily on which days), so that professional and patient can assess afterwards whether the activity has been completed or not. The task gets documented for the patient, e.g. written on a piece of paper that the patient takes along.

How are the steps covered during the sessions?

How much time each step takes and to what extent they are covered in one or two sessions varies.

Usually, the first meeting would end with steps 5 or 6, and after the second meeting an activity is agreed upon. However, it is allowed that patients and professional leave the decision to a third session or take it during the first session if this is possible.

During follow-up sessions, social coach and patient discuss to what extent the activity has been done. The social coach provides positive feedback and – if required – deals with complete or partial perceived failure using solution focused techniques (e.g. emphasising what went well). In case professional and patient come to the conclusion that the originally planned activity does not work, a face-to-face meeting is arranged in which steps 4 to 8 of the initial meetings are repeated and a different activity is planned. In most cases, this is done before the first activity has been tried for a three months.

BMJ Open

Effectiveness and cost-effectiveness of a structured social coaching intervention for people with psychosis (SCENE): trial protocol

Journal:	*BMJ Open*
Manuscript ID	bmjopen-2021-050627.R1
Article Type:	Protocol
Date Submitted by the Author:	29-Sep-2021
Complete List of Authors:	Giacco, Domenico; University of Warwick; Newham Centre for Mental Health, Unit for Social and Community Psychiatry<br>Chevalier, Agnes; Newham Centre for Mental Health, Unit for Social and Community Psychiatry<br>Patterson, Megan; Newham Centre for Mental Health, Unit for Social and Community Psychiatry<br>Hamborg, Thomas; Queen Mary University of London<br>Mortimer, Rianna; Queen Mary University of London<br>Feng, Yan; Queen Mary University of London, Blizard Institute<br>Webber, Martin; University of York, Department of Social Policy and Social Work<br>Xanthopoulou, Penny; University of Exeter, Medical School<br>Priebe, Stefan; Barts and the London School of Medicine and Dentistry, University of London, Unit of Social and Community Psychiatry
**Primary Subject Heading**:	Mental health
Secondary Subject Heading:	Evidence based practice, Health economics, Health services research, Rehabilitation medicine
Keywords:	Schizophrenia & psychotic disorders < PSYCHIATRY, SOCIAL MEDICINE, MENTAL HEALTH

SCHOLARONE™
Manuscripts

I, the Submitting Author has the right to grant and does grant on behalf of all authors of the Work (as defined in the below author licence), an exclusive licence and/or a non-exclusive licence for contributions from authors who are: i) UK Crown employees; ii) where BMJ has agreed a CC-BY licence shall apply, and/or iii) in accordance with the terms applicable for US Federal Government officers or employees acting as part of their official duties; on a worldwide, perpetual, irrevocable, royalty-free basis to BMJ Publishing Group Ltd ("BMJ") its licensees and where the relevant Journal is co-owned by BMJ to the co-owners of the Journal, to publish the Work in this journal and any other BMJ products and to exploit all rights, as set out in our licence.

The Submitting Author accepts and understands that any supply made under these terms is made by BMJ to the Submitting Author unless you are acting as an employee on behalf of your employer or a postgraduate student of an affiliated institution which is paying any applicable article publishing charge ("APC") for Open Access articles. Where the Submitting Author wishes to make the Work available on an Open Access basis (and intends to pay the relevant APC), the terms of reuse of such Open Access shall be governed by a Creative Commons licence – details of these licences and which Creative Commons licence will apply to this Work are set out in our licence referred to above.

Other than as permitted in any relevant BMJ Author's Self Archiving Policies, I confirm this Work has not been accepted for publication elsewhere, is not being considered for publication elsewhere and does not duplicate material already published. I confirm all authors consent to publication of this Work and authorise the granting of this licence.

For peer review only

Effectiveness and cost-effectiveness of a structured social coaching intervention for people with psychosis (SCENE): trial protocol

4236 words

Authors:

Domenico Giacco,[1,2] Agnes Chevalier,[2] Megan Patterson,[2] Thomas Hamborg,[3] Rianna Mortimer,[3] Yan Feng,[3] Martin Webber,[4] Penny Xanthopoulou,[5] Stefan Priebe.[2]

Affiliations:

[1] Division of Health Sciences, Warwick Medical School, University of Warwick, Coventry, CV4 7AL

[2] Unit for Social and Community Psychiatry, (WHO Collaborating Centre for Mental Health Service Development), Barts and the London School of Medicine, Queen Mary University of London, Newham Centre for Mental Health, London, E13 8SP

[3] Institute of Population Health Sciences, Barts and the London School of Medicine, Queen Mary University of London, Yvonne Carter Building, 58 Turner Street, London E1 2AB, UK.

[4] International Centre for Mental Health Social Research, Department of Social Policy and Social Work, University of York, Heslington, York, YO10 5DD, UK.

[5] College of Medicine and Health, University of Exeter. St Luke's Campus, Exeter, EX2 4TH, UK.

Correspondence to Dr Domenico Giacco; domenico.giacco@warwick.ac.uk

For peer review only

ABSTRACT (299 words)

Introduction

People with psychosis tend to have smaller social networks than both people in the general population and other people with long-term health conditions. Small social networks are associated with poor quality of life. Preliminary evidence suggests that coaching patients to increase their social contacts may be effective. In this study we assessed whether structured social coaching improves the quality of life of patients with psychosis (primary outcome) compared to an active control group, receiving information on local social activities.

Methods and analysis

A structured social coaching intervention was developed based on the literature and refined through stakeholder involvement. It draws on principles from motivational interviewing, solution focused therapy and structured information-giving. It is provided over a six month period and can be delivered by a range of different mental health professionals. Its effectiveness and cost-effectiveness are assessed in a randomised controlled trial, compared to an active control group, in which participants are given an information booklet on local social activities.

Participants are aged 18 or over, have a primary diagnosis of a psychotic disorder (International Classification of Disease: F20-29) and capacity to provide informed consent. Participants are assessed at baseline and at six, 12 and 18 months after individual randomisation. The primary outcome is quality of life at six months (Manchester Short Assessment of Quality of Life). We hypothesise that the effects on quality of life are mediated by an increase in social contacts. Secondary outcomes are symptoms, social situation and time spent in social activities. Costs and cost-effectiveness analyses will consider service use and health-related quality of life.

Ethics and dissemination

NHS REC London Hampstead [19/LO/0088] provided a favourable opinion. Findings will be disseminated through a website, social media, scientific papers and user-friendly reports, in collaboration with a lived experience advisory panel.

Registration

ISRCTN registry (15815862), https://doi.org/10.1186/ISRCTN15815862

For peer review only

SUMMARY

Strengths

- Broad inclusion criteria, allowing inclusion of patients with a range of individual characteristics (e.g. varying level of financial difficulties, and different types and levels of symptoms).
- Inclusion of a large number of urban, semi-urban and rural sites, as different geographical contexts may influence the availability or convenience of social activities
- Active control condition, i.e. the provision of comprehensive information on local social activities.

Limitations

- During this trial we focused on people of working age (18-65) and only included people who are fluent in English.
- Since social coaching was not part of routine care before this trial, the coaches in the experimental intervention are – although trained – not experienced in this type of approach.

For peer review only

INTRODUCTION

At any given time, more than 200,000 people experience a psychotic disorder in England alone. The total costs for England was estimated to be £4 billion in 2007 and £6.5 billion by 2026.[1] People with psychotic disorders have much smaller social network sizes compared to the general population, and compared to other groups with long-term mental and physical health problems.[2-3] Social isolation is not only a serious problem in itself, but is also linked with poor quality of life and a range of unfavourable health oucomes.[3-6]

Traditionally, pharmacological and psychological treatments have attempted to reduce the social isolation of patients with psychosis indirectly; through treating symptoms or by teaching social skills.[7] However, the symptoms of psychosis which are mostly linked with social isolation, i.e. the “negative symptoms” do not show a substantial response to established pharmacological treatments.[8] Social skills training has been found to be effective in teaching these skills to patients, however this does not translate to improved social functioning.[9] Given the evidence that its benefits are limited, social skills training is not recommended by NICE guidelines.[4]

A systematic review by Anderson et al.[7] found that interventions which directly focused on supporting socialisation activities had a positive effect on reducing social isolation.[10-13] These interventions were diverse, including guided peer support, social coaching, and dog-assisted integrative psychological therapy. The largest and highest quality trial among the ones identified tested a social coaching intervention, which was the only intervention clearly targeting social contacts outside of services [11]. This led to the decision that this model would inform our intervention development. One of the limitations identified by the systematic review was that none of the studies reported an economic analysis of the costs and benefits of the interventions [7].

In a research programme funded by the National Institute for Health Research (NIHR) (https://scene.elft.nhs.uk), we developed a manualised and structured social coaching intervention. The effective components identified in the international literature[7] were refined and adapted to the English NHS context. The intervention was designed to improve patients' quality of life through increasing their social networks.

OBJECTIVES

Primary objective

To assess whether the structured social coaching intervention improves the quality of life of patients with psychosis (primary outcome) compared to an active control group, which received information on local social activities.

Secondary objectives

1. To understand whether changes in quality of life are mediated by an increase in social contacts (in the previous week).

For peer review only

2. To evaluate whether the intervention improves secondary outcomes such as social contacts, symptoms, social situation, feelings of loneliness, time spent in social activities, health-related quality of life and whether it reduces service use.
3. To assess costs and cost-effectiveness of the intervention.
4. To explore implementation of the intervention.

METHODS

Study design

Individually randomised, parallel group controlled trial. The intervention and control condition are provided in addition to standard care.

Study sites

This multi-centre study is led and sponsored by East London NHS Foundation Trust (https://www.elft.nhs.uk/Contact-Us) and includes the sites listed in Table 1. The study is currently open to additional sites.

[Insert Table 1 here]

Eligibility criteria

Inclusion criteria

Patients:

- 18-65 years old
- Diagnosis of psychosis-related condition (ICD-10 F20-29)
- Capacity to provide informed consent
- Ability to communicate in English
- Limited social network size (three or less social contacts with non-first degree relatives in the previous week)
- Low quality of life (Score 5 or less on the MANSA quality of life assessment)
- Not receiving hospital treatment at the time of recruitment

Social coaches:

- Mental health professionals with experience of providing mental health care (e.g. psychiatrists, clinical psychologists, nursing staff, occupational therapists), minimum NHS Band 4 or equivalent experience.
- Aged 18 and over
- Capacity to provide informed consent
- Ability to communicate in English

Intervention development

For peer review only

The intervention developed by Terzian et al.[11] was taken as the starting point for the intervention tested in this trial. It was specified and expanded using approaches from solution-focused therapy and motivational interviewing, and considering previous experiences of the group in developing and evaluating complex psycho-social interventions, in particular DIALOG+ [14]. It was further modified and then manualised by the Programme Management group, which included experts in psychiatry, psychology, social work, occupational therapy, social sciences and behavioural change and experts by experience. The intervention was finalised in consultation with stakeholders.[15] The intervention is considered to be generic and not profession-specific, so that it can be delivered by different professional groups. The role of the social coach is intended to be independent of other treatment and solely focused on the task of expanding social networks. Social coaches are not meant to establish a wider or longer-term therapeutic relationship, which might interfere with other therapeutic relationships of the patients. The theoretical framework used is shown in Figure 1.

[Insert Figure 1 here]

Randomisation procedures

Patients are individually randomised to either the intervention or control group. The allocation ratio is 1:1. Randomisation is stratified by site (NHS Trust), ensuring balanced numbers of patients in each group at each NHS Trust. Permuted blocked randomisation with block sizes of m=6, 4 and 2 are used within each stratum. Patients are allocated to clinicians based on locality and availability i.e. not randomly.The randomisation is carried out remotely by the Pragmatic Clinical Trials Unit at Queen Mary, University of London, which is also responsible for database development and assists the team with data monitoring.

Trial arms

<u>Arm 1. Intervention</u>

a. *Who delivers the intervention*

The intervention is delivered by clinicians from different backgrounds (e.g. psychologists/assistant psychologists, social workers, nursing staff, occupational therapists and medical doctors), with a minimum level of experience and seniority equivalent to a NHS band 4. They take up a role of “social coach” for the treated patient.

b. *Type and frequency of sessions*

Social coaches meet patients at least three times but ideally monthly over the six-month intervention period. During the first two sessions of the intervention, a structured eight step approach is followed and then revisited in follow-up meetings. Please see details of the eight step approach and of the intervention sessions in Box 1.

[Insert box 1 here]

The intervention starts with two initial face-to-face sessions (which can also occur via video conferencing), each lasting between 60 and 90 minutes. The main aim of these initial meetings is to introduce the intervention, explore participants’ social history and discuss preferences

For peer review only

and options for activities. The participant then selects one social activity to focus on during the remaining meetings and actions are agreed. The subsequent meetings include discussions around challenges and progress and and take place monthly, lasting about 20 minutes each. The final meeting is face-to-face and is used as both a summary of progress and to plan for future social activities after the intervention. The intervention can be stopped at any time on participant request.

c. Training and supervision of social coaches

Social coaches are trained in one session lasting three hours, normally in a group format (although one-to-one sessions can be arranged). Training is provided by senior SCENE researchers.

During the training coaches acquire knowledge of the structure and aims of the intervention, and of simple motivational interviewing (e.g. identifying change talk) and solution focused therapy techniques (e.g. identifying what has worked in the past). Scenarios in which barriers for the patients in engaging in new social activities may appear and strategies to overcome them are discussed.

Learning progress is assessed during the training and in the subsequent supervision, provided by senior members of the research team.

Clinicians receive updates on changes in options for activities from the local research team. They will also receive at least two supervision sessions by SCENE senior researchers.

<u>Arm 2. Control group</u>

Patients in the control group are provided with information about local options for social activities via a booklet sent to them via post or handed over by a researcher. This is intended to control for the provision of information on social activities and service attention to their social isolation.

Outcomes

<u>Primary Outcome</u>

The primary outcome is subjective quality of life, measured with the Manchester Short Assessment of Quality of Life (MANSA) at the end of the intervention period (6 months after randomisation).

The MANSA has been widely used in research and cited in more than 850 research papers. The MANSA is brief with very high completion rates and excellent psychometric properties.[16]

<u>Mediator of effect on primary outcome</u>

Number of social contacts in the previous week, measured at 6 months follow-up using the Social Contacts Assessment (SCA)[17-18].

For peer review only

Secondary outcomes

- Social Contacts (SCA).[17]
- Psychopathological symptoms assessed with the Positive And Negative Syndrome Scale (PANSS).[19]
- Social situation with the SIX.[20]
- Feeling of loneliness with the UCLA Loneliness Scale.[21]
- Time spent in social activities with the Time Use Survey.[22]
- Health-related quality of life with the EQ-5D-5L.[23]
- Service use with the Client Service Receipt Inventory,[24] and from NHS Digital datasets.[25]

Study outcomes and timepoints are summarised in Table 2.

[Insert Table 2 here]

Patient and public involvement

A Lived Experience Advisory Panel (LEAP) has been set-up and meets every six months to advise on study progress, review materials and support dissemination plans.

The LEAP has a central role in the preparation of study materials, design of practical procedures, and dissemination. For example, LEAP members provided valuable feedback during the development of the intervention, and for facilitating recruitment and finding out about available activities in the community. The chair of the LEAP is an expert by experience who attends regular meetings with the project team and is directly involved in parts of the research, in particular the interpretation of qualitative data. Findings from all work packages, including the intervention development are discussed with and influenced by the LEAP.

Internal pilot

The trial comprised an internal pilot with the aim of checking the feasibility of recruiting to target. The recruitment target for the internal pilot was 140 participants representing an average rate of four participants per site per month for five months. This was achieved within the 5 month time frame.

Trial registration

The trial was registered with the ISRCTN (15815862). Whilst listed restrospectively, the initial request for registration was made on the 27/02/2019, ahead of the recruitment start date and thus considered to have been prospectively registered according to ICMJE guidelines.

Independent Committees

For peer review only

The trial has an independent Project Steering Committee and a Data Monitoring Committee. Both include among their members one clinician/clinical researcher, one quantitative methodologist and one person with lived experience of mental illness.

ANALYSES

Statistical Analysis

The primary outcome analysis will be the comparison of mean MANSA scores between treatment groups at 6 months follow-up using a heteroscedastic partially nested mixed-effects model.[26] This model will account for clustering by treating clinician in the intervention arm, baseline values of the outcome (MANSA) and *site* as covariates.

Secondary outcomes will be analysed using the same model as for the primary outcome or an equivalent model appropriate for the outcome type where the secondary outcome is not continuous. Differences in outcome measures between groups will be compared for 6 month, 12 month and 18 month follow-up data. Additionally, repeated measures models comprising all four time points will be fitted. Baseline characteristics of patients will be tabulated by treatment arms.

The analysis will be on an intention-to-treat basis, and every effort will be made to collect complete data. If any outcome data are missing, available subject data only will be analysed (unbiased analysis under missing-at-random assumption); however, patterns of missing data will be explored, and a strategy for dealing with missing values will be articulated in the formal statistical analysis plan.

We are planning individual level single imputations (replacing missing values by a fixed value defined by a certain rule) analyses for partially completed primary outcome data to assess the uncertainty around the primary outcome analysis estimate. Further details of other sensitivity analyses planned will be outlined in the statistical analysis plan prior to analysis.

A mediator analysis will identify whether the effect on the primary outcome is mediated through expanded social networks (SCA) at six months, as hypothesised. The product of coefficients method, with a Sobel test and bootstrap standard errors will be used.[27] Further mediation analyses will assess the mediation effect of increases in SCA at 6 months on patients' MANSA score at 12 month follow-up.

All analyses are incorporated into a statistical analysis plan, and allocation codes will not be released to the statistician before the analysis plan is signed off. All researchers involved in developing the analysis plan will remain blinded until the analysis plan is signed off.

<u>Sample size calculation</u>

It is assumed that the proposed new intervention would be implemented and funded across the NHS only if it achieved at least a medium sized effect. An effect size of 0.35 is equivalent to an improvement of satisfaction ratings on the MANSA of at least one scale point (on a 7

For peer review only - http://bmjopen.bmj.com/site/about/guidelines.xhtml

point scale) for 4 out of a total of 12 life domains. An improvement of quality of life in 4 life domains is usually regarded as a meaningful difference to patients' life.[16]
For detecting such an effect size with 90% power, assuming a conservative ICC of 0.07 for patients treated by the same professional in the intervention group, 229 patients in the intervention group and 229 in the control group will be required (total sample = 458). A 1:1 allocation ratio has been chosen for organisational ease. This requires 8 additional patients to be recruited compared to the absolute minimum required sample size with slightly uneven groups. A drop-out rate (from the study) at 6 months follow-up of 20% (in line with recent trials of similar interventions with the same patient group) (VOLUME trial) was assumed.[18] The sample size calculation was based on 10 patients being treated and followed-up per clinician on average. Based on recruiting 12 patients per clinician the final total sample size target was 576 patients (288 per arm). Recruitment and intervention delivery to SCENE were paused during the Covid-19 pandemic making a study extension necessary (see section IMPACT OF THE COVID PANDEMIC). For this a sample size re-calculation was conducted using values observed so far for two quantities. The dropout rate was inflated from 20% to 25% (actual rate to date 24%). Cluster size in the intervention arm was reduced from 10 to 3 patients per treating professional on average allowing for observed variability in patients per professional. Using these estimates the updated total number of patients to recruit is 504.

Qualitative process evaluation

A qualitative process evaluation will be conducted, employing semi-structured interviews with a number of purposively selected patients and social coaches. Interviews will be transcribed and analysed using thematic analysis.[28]

We will interview 40 patients in the experimental group to explore experiences of the intervention and descriptions of qualitative changes in their social network, contacts and activities. A topic guide for interviews was developed with input from the LEAP. Interviews will be conducted after the end of the six-month outcome assessment, so that the interviews do not interfere with the effects of the intervention in influencing the primary outcome (quality of life at six months). Un-blinded researchers will identify particpants, conduct the interviews and manage qualitative data, so researchers assessing outcomes remain blind to allocation
We will use purposive sampling, to include patients who differ according to gender, whether they live in urban or rural settings andwhether or not they completed the intervention. Sampling of social coaches will include those who have seen more than three patients and those who have seen fewer.

Adherence to manual

Adherence to manual will be assessed through our adherence checklist. Routine documentation and audiotapes of patient-professional meetings (for consenting participants) will be compared against the clinician-reported adherence schedule to check reliability. This is a self-reported checklist of whether and how the different steps of the intervention (Box 1) are addressed. Clinicians will have addressed all the eight steps of the intervention and conducted at least three sessions with a given patient for the intervention to be deemed as completed.

For peer review only

Economic evaluation

The within trial analysis will adopt the NHS and Personal Social Services perspective to assess the cost-effectiveness of the psychosocial intervention for patients with psychosis compared to best standard practice.[29] The evaluation will focus on the 18 months from baseline until the end of the follow-up period. The analysis will adhere to guidelines for good economic evaluation practice as outlined in Ramsey et al.[30] and Sanders et al.[31]

Resource-use associated with delivery of the interventions in both trial arms will be identified using a specially designed intervention implementation form. Participants' use of health services, including mental health and hospital care, will be extracted from the NHS Digital database. All participants will be asked to complete a modified version of the Client Services Receipt Inventory to obtain information about their of other psychosocial interventions, medication and receipt of informal care from families and friends. The quantities of all resource use will be combined with unit costs to generate cost at the individual participant level. Unit costs will be obtained from various sources, including a specially designed coach demographics questionnaire, NHS Reference costs,[32] Unit Costs of Health and Social Care,[33] NHS drug Tariff,[34] and the UK earnings data.[35]

As for outcomes, the primary outcome measure for the economic evaluation will be collected using the EQ-5D-5L instrument.[23] We will calculate the participant level Quality Adjusted Life Years (QALYs) and use the EQ-5D-5L data with the area-under-the-curve approach.[36] The secondary outcome will be measured using the MANSA. The time points for collecting costs and outcome data are reported in Table 2.

Both costs and outcomes occurring during the last 6 months of the follow-up period will be discounted at 3.5% in line with the NICE recommendation.[29] Costs and outcome data will be analysed by treatment allocation, and differences between trial arms will be estimated over 18-months, adjusting for baseline differences using regression analysis. We will select an appropriate method to handle missing data based on the nature of our data.

Cost-utility analysis and cost-effectiveness analysis will be applied in the economic evaluation. In the cost-utility analysis, the estimates of incremental cost-effectiveness ratio (ICER) for the psychosocial intervention compared to best standard practice will be presented against the decision maker's willingness to pay a value of £20,000 to £30,000 per QALY.[23] To report the uncertainty of the point estimate of ICER, we will use the non-parametric bootstrap approach to estimate the confidence interval around the ICER. We will also present the probability that the intervention is cost-effective against a range of decision makers' willingness to pay value using the cost effectiveness acceptability curve. In the cost-effectiveness analysis, we will calculate the incremental cost per unit change on the MANSA scale and uncertainty surrounding the ratio.

A number of sensitivity analyses to assess the impact of key assumptions as well as uncertainty with key parameters in the economic evaluation will be conducted to (1) explore the impact of alternative assumptions about the missing data mechanism; (2) consider uncertainty in the most important cost drivers to assess the impact of healthcare use; (3) use

For peer review only

a broader analytical perspective by including additional costs for informal care; (4) use 0 to 6 months from randomisation as the time period for economic evaluation.

ETHICS AND DISSEMINATION

The study was reviewed and a favourable opinion received from the London Hampstead NHS Research Ethics Committee [19/LO/0088]. Any serious adverse events are recorded in specific forms and their relationship with the intervention are adjudicated by site leads who are all senior clinicians. Written informed consent is provided by participants after discussion with researchers. A model consent form is enclosed as Supplementary Document.

Any personal information stored in locked cabinets on NHS premises if in paper version, and encrypted if in electronic version. Dataset will be accessed by the study team and after the primary analysis may be made available to other parties subject to data sharing agreements.

Throughout all phases of the research, we will disseminate information about the activities of the programme through social media and a project specific website (http://scene.elft.nhs.uk/) in order to reach a wider public audience. Authorship guidelines for outputs will follow the ICMJE guidelines.

When results become available, they will be disseminated through:

- scientific publications in peer-reviewed open-access journals;
- presentations at national and international conferences and to professional and non-professional audiences at appropriate events;
- existing networks, including but not limited to the World Health Organisation, the benchmarking network in mental health, organisations involved in Quality Improvement programmes and the professional networks of the programme management group members.

IMPACT OF THE COVID PANDEMIC

Following the outbreak of COVID-19 in the United Kingdom, recruitment was stopped from 16/3/2020 to 01/10/2020. We decided to stop the intervention delivery from 16/3/2020 to 21/8/2020 in accordance with social distancing guidelines at that time.

We added an additional follow-up at 10 months from randomisation for those participants who had already been randomised when the study was stopped, but had not completed the treatment period. A sensitivity analysis will consider end-of-treatment as the outcome of comparison, and hence include the assessment of quality of life at 10 months from randomisation rather than at 6 months for these participants.

We created additional adapted versions (used separately from the standard ones) of the Social Contact Assessment and of the Time Use Survey and data collected will be analysed to capture online social contacts and activities throughout the trial.

The recruitment and randomisation of participants was resumed on 01/10/2020. Additional instructions to social coaches were provided on physical distancing with patients and on how to encourage social activities which are either online or can be carried in accordance with different scenarios and different physical distancing directives.

For peer review only

Research follow-up of participants at different timepoints was never stopped and continued over the phone or via video-conferencing.
We have calculated that 70 participants may have been especially affected by the COVID-19 pandemic in that their primary outcome was assessed at a time when restrictions meant that they could not meet more than one person outside of their household.
To adjust for any pandemic effects on the intervention itself, the outcomes or both, a sensitivity analysis will adopt a mixed-effects model approach, grouping participants according to the physical distancing guidance that they have been exposed to. Individual treatment effect estimates of participant groups with different levels of exposure will be calculated.

DISCUSSION

This study addresses a gap in mental health care provision, i.e. the lack of treatments available to help patients overcome their social isolation[4]. It has broad inclusion criteria and is carried out across a large number of urban, semi-urban and rural sites. This will allow to control by design a number of patient-level (e.g. financial and clinical status) and area-level characteristics (e.g. availability of affordable activities, distance and travel required to access them) which might influence intervention effectiveness. The active control condition, i.e. the provision of comprehensive information on local social activities, does not only control for service attention to social activity and information provision, but also arguably represents a reflection of best current practice.

The methodological choices made when designing the study come with some limitations:

- Older people and those who are not fluent in the language of their country of residence encounter additional barriers to socialisation. Hence, we restricted inclusion criteria to patients with psychotic disorders of working age and to those who are fluent in English, to limit heterogeneity of our sample. Future trials and/or implementation studies should target these populations specifically.
- The new intervention has been implemented in services directly as part of the trial. Social coaches are trained but do not have previous experience in delivering this type of approach.

In addition to this, the COVID-19 outbreak meant that more of the intervention has to be delivered remotely than originally envisaged, and we do not know whether and how that will impact on outcomes. We have a sensitivity analysis to estimate this as explained in detail in the following paragraph.

If the intervention is found to be effective in increasing social contacts and improving quality of life, it can promptly become part of the therapeutic armamentarium of mental health services. The flexibility of the approach, which can be delivered by different type of professionals, might facilitate its uptake within the ever evolving landscape of mental health services. In line with the design of the trial, we do not intend for the social coach to become be a new professional rolein itself. Instead, the function of social coaches can be taken on by different professionals and exercised along with other clinical activities.

For peer review only

AUTHORS' CONTRIBUTIONS

Giacco led the study protocol development and is the Chief Investigator. Priebe led the intervention development and is the programme co-lead. Mortimer and Hamborg led the statistical analysis plans. Feng led the health economy analysis section. Chevalier, Patterson, Webber and Xanthopoulou contributed to intervention development and study protocol development. All authors provided intellectual input to the paper and approved its final version.

FUNDING STATEMENT

This research is funded by the National Institute for Health Research Programme Grants for Applied Research funding stream (award number: RP-PG-0615-20009). This article reflects views of the authors and not necessarily those of the NIHR. The NIHR and the study sponsor (Eats London NHS Foundation Trust) had no role in in study design, data collection, management or analysis, and interpretation of data or in the writing of the report or the decision to submit the report for publication.

COMPETING INTERESTS STATEMENT

The authors declare no competing interests.

REFERENCES

1 McCrone P, Dhanasiri S, Patel A, et al. Paying the Price: The Cost of Mental Health Care in England to 2026. King's Fund 2008. https://www.kingsfund.org.uk/sites/default/files/Paying-the-Price-the-cost-of-mental-health-care-England-2026-McCrone-Dhanasiri-Patel-Knapp-Lawton-Smith-Kings-Fund-May-2008_0.pdf [accessed Feb 2021]

2 Palumbo C, Volpe U, Matanov A, et al. Social: networks of patients with psychosis: a systematic review. *BMC Res Notes* 2015;8:560.

3 Degnan A, Berry K, Sweet D, et al. Social networks and symptomatic and functional outcomes in schizophrenia: a systematic review and meta-analysis. *Soc Psychiatry Psychiatr Epidemiol* 2018; 53(9):873-888.

4 National Institute for Health and Care Excellence (NICE). Psychosis and Schizophrenia in Adults: prevention and management 2014; [CG178]. https://www.nice.org.uk/guidance/cg178/evidence/full-guideline-490503565 [accessed Feb 2021]

5 Bengtsson-Tops A, Hansson L. Quantitative and qualitative aspects of the social network in schizophrenic patients living in the community. Relationship to sociodemographic characteristics and clinical factors and subjective quality of life. *International Journal of Social Psychiatry* 2001;47(3):67-77.

6 Norman R.M, Malla A.K, Manchanda R, et al. Social support and three-year symptom and admission outcomes for first episode psychosis. *Schizophrenia Research* 2005; 80(2-3):227-34.

7 Anderson K, Laxhman N, Priebe S. Can mental health interventions change social networks? A systematic review. *BMC Psychiatry (Open Access)* 2015; 15:297.

For peer review only

8 Fusar-Poli P, Papanastasiou E, Stahl D, et al. Treatments of Negative Symptoms in Schizophrenia: Meta-Analysis of 168 Randomized Placebo-Controlled Trials. *Schizophr Bull* 2015; 41(4):892-9.
9 Almerie M.Q, Okba Al Marhi M, Jawoosh M, et al. Social skills programmes for schizophrenia. *Cochrane Database of Systematic Reviews* 2015; 2015(6):CD009006
10 Castelein S, Bruggeman R, Van Busschbach JT, et al. The effectiveness of peer support groups in psychosis: A randomized controlled trial. *Acta Psychiatr Scand* 2008;118:64–72
11 Terzian E, Tognoni G, Bracco R, et al. Social Network Intervention in Patients With Schizophrenia and Marked Social Withdrawal: A Randomized Controlled Study. *Can J Psychiatry* 2013;58:622–631.
12 Sheridan AJ, Drennan J, Coughlan B et al., Improving social functioning and reducing social isolation and loneliness among people with enduring mental illness: Report of a randomised controlled trial of supported socialisation. *Int J Soc Psychiatry* 2014;61(3):241-50.
13 Hasson-Ohayon I, Mashiach-Eizenberg M, Avidan M, et al. Social Cognition and Interaction Training: Preliminary Results of an RCT in a Community Setting in Israel. *Psychiatr Serv* 2014;65:555–558.
14 Giacco D, Priebe S. Using Routine Quality of Life Assessment to Improve Effectiveness of Community Mental Health Care. In: Awad AG, Voruganti LNP, eds. Beyond Assessment of Quality of Life in Schizophrenia. Springer International Publishing Switzerland 2016:145-155.
15 Tee H, Priebe S, Santos C, et al. Helping people with psychosis to expand their social networks: the stakeholders' views. *BMC Psychiatry* 2020;20(1):29.
16 Priebe S, Kellye L, Omer S, et al. The Effectiveness of a Patient-Centred Assessment with a Solution-Focused Approach (DIALOG+) for Patients with Psychosis: A Pragmatic Cluster-Randomised Controlled Trial in Community Care. *Psychotherapy and Psychosomatics* 2015;84:304-313.
17 Giacco D, Palumbo C, Strappelli N, et al. Social contacts and loneliness in people with psychotic and mood disorders. *Compr Psychiatry* 2016;66:59-66.
18 Priebe S, Chevalier A, Hamborg T, et al. Effectiveness of a volunteer befriending programme for patients with schizophrenia: randomised controlled trial. The British Journal of Psychiatry. Cambridge University Press; 2020;217(3):477–83.
19 Kay S. Positive and Negative Syndromes in Schizophrenia. New York: Brunner/Mazel 1991.
20 Priebe S, Watzke S, Hansson L, et al. Objective social outcomes index (SIX): a method to summarise objective indicators of social outcomes in mental health care. *Acta Psychiatr Scand* 2008;118(1):57-63.
21 Hays R.D, DiMatteo M.R. A short-form measure of loneliness. *Journal of personality assessment* 1987;51(1):69-81.
22 Priebe S, Pavlickova H, Eldridge S, et al. Effectiveness of one-to-one volunteer support for patients with psychosis: protocol of a randomised controlled trial. *BMJ open* 2016;6(8).
23 Herdman M, Gudex C, Lloyd A, et al. Development and preliminary testing of the new five-level version of EQ-5D (EQ-5D-5L) *Qual Life Res* 2011;20:1727–36.
24 Beecham J, Knapp M. Costing psychiatric interventions. In: Thornicroft G, eds. Measuring Mental Health Needs. Gaskell, 2nd edition 2001:200-224.

For peer review only

25 NHS Digital. Secondary Uses Service (SUS). Available from https://digital.nhs.uk/services/secondary-uses-service-sus [accessed Feb 2021].
26 Candlish J, Teare M.D, Dimairo M, et al. Appropriate statistical methods for analysing partially nested randomised controlled trials with continuous outcomes: a simulation study. *BMC Med Res Methodol* 2018;18:105.
27 Fritz CO, Morris PE, Richler JJ. Effect size estimates: current use, calculations, and interpretation. *J Exp Psychol Gen* 2012;141(1):2-18.
28 Braun V, Clarke V. Using thematic analysis in psychology. *Qualitative Research in Psychology* 2006;3(2):77–101.
29 National Institute for Health and Care Excellence (NICE). Guide to the methods of technology appraisal 2013; https://www.nice.org.uk/process/pmg9/resources/guide-to-the-methods-of-technology-appraisal-2013-pdf-2007975843781 [accessed Aug 2020]
30 Ramsey S.D, Willke R.J, Glick H, et al. Cost-effectiveness analysis alongside clinical trials II-an ISPOR good research practices task force report. *Value Health* 2015;18(2):161–172.
31 Sanders GD, Neumann PJ, Basu A, et al. Recommendations for conduct, methodological practices, and reporting of cost-effectiveness analyses: second panel on cost-effectiveness in health and medicine. JAMA 2016;316(10):1093–1103.
32 NHS improvement. National Cost Collection for the NHS. https://www.england.nhs.uk/national-cost-collection/ [accessed Aug 2020]
33 Curtis L, Burns A. Unit Costs of Health and Social Care 2019; https://www.pssru.ac.uk/project-pages/unit-costs/unit-costs-2019/ [accessed Aug 2020]
34 NHS Business Service Authority. Drug Tariff online. https://www.nhsbsa.nhs.uk/pharmacies-gp-practices-and-appliance-contractors/drug-tariff [accessed Aug 2020]
35 Office for National Statistics. Earnings and working hours. https://www.ons.gov.uk/employmentandlabourmarket/peopleinwork/earningsandworkinghours [accessed Aug 2020]
36 Matthews JN, Altman DG, Campbell MJ, et al. Analysis of serial measurements in medical research. *BMJ* 1990;300:230–235

For peer review only

Table 1. Trial sites

East London NHS Foundation Trust (two sites: East London and Luton)
Tees, Esk and Wear valleys NHS Foundation Trust
Devon Partnership NHS Trust
Cornwall NHS Partnership NHS Trust
Oxford Health NHS Foundation Trust
Somerset NHS Foundation Trust
Leeds and York NHS Foundation Trust
Humber Taching NHS Foundation Trust
Gloucestershire Health and Care NHS Foundation Trust
Coventry and Warwickshire Partnership NHS Trust
Lincolnshire Partnership NHS Trust

For peer review only

Table 2. SCENE Study outcomes and timepoints.

Assessment	Screening	Baseline	Study phase (6 months)	Follow up (12 months)	Follow up (18 months)
All Patient Participants					
MANSA	x	x	x	x	x
Social Contacts Assessment	x	x	x	x	x
PANSS		x	x	x	x
Social situation		x	x	x	x
Loneliness		x	x	x	x
Time spent in social activities		x	x	x	x
EQ-5D-5L		x	x	x	x
Client Service Receipt Inventory		x	x	x	x
Healthcare source use (NHS Digital)		x	x	x	x
Intervention Participants only					
Semi-structured interviews			x		
Social coaches					
Adherence schedule			x		
Semi-Structured Interviews			x		

For peer review only

Box 1. SCENE Social coaching intervention: the eight step approach

1) Introduction: The social coach and the patient introduce themselves.

2) Clarification of the remit of the intervention: The professional explains and discusses the focused remit of the intervention, i.e. that it aims to expand social networks and that all other therapeutic issues have to be addressed elsewhere.

3) Exploration of past and current activities: The social coach explores past activities that involved social contacts; this should be done chronologically covering the adult life time of the patient from the age of 15 years onwards, and stepwise for periods of 5 – 10 years. At the end of the exploration, professional and patient go through the list of activities (if any) and discuss to what extent the patient enjoyed each activity. Solution focused therapy techniques (e.g. identifying what went well and wat worked) can be used.

4) Motivation for change: The professional explores and discusses the patient's motivation to change and expand their social networks. Motivational interviewing techniques (e.g. identifying change talk) can be used.

5) Options for activities: Professional and patient discuss which new activities (or expanding existing ones respectively) the patient considers.

6) Information: The professional provides as much helpful information as possible about options in the given locality for patients' preferred activities. Professional and patient discuss the practicalities and sometimes decide to obtain further information. In this step, the patient is encouraged and supported to find information him/herself. Yet, if this is a substantial hurdle, the professional provides as much direct support as needed.

7) Consideration and decision: Once options have been identified, the patient is asked to consider taking it up. If the patient is ambivalent, patients are encouraged to take time, e.g. a week until the next meeting or a phone call, to think about it.

8) Definition of activity: Finally, the patient decides on the type of activity and some specification of the actual steps (e.g. twice per week attending a certain class, but not necessarily on which days), so that professional and patient can assess afterwards whether the activity has been completed or not. The task gets documented for the patient, e.g. written on a piece of paper that the patient takes along.

How are the steps covered during the sessions?

There is some flexibility as to how much time each step takes and to what extent they are covered within the monthly sessions varies.

Session 1: Usually, the first meeting would end with steps 5 or 6.

Session 2: After the second meeting an activity is agreed upon. However, it is allowed that patients and professional leave the decision to a third session or take it during the first session if this is possible.

Session 3-6 (follow-up sessions): During follow-up sessions, social coach and patient discuss to what extent the activity has been done. The social coach provides positive feedback and – if required – deals with complete or partial perceived failure using solution focused techniques (e.g. emphasising what went well).

After three months (i.e. session 3-4), the situation is re-evaluated. If the activity is working well, then this is monitored in further follow-up sessions. In case professional and patient come to the conclusion that the originally planned activity does not work, a face-to-face meeting is arranged in which steps 4 to 8 of the initial meetings are repeated and a different activity is planned.

FIGURE LEGEND

Figure 1. Theoretical model of intervention processes

For peer review only

Adapted from the iterative model of self regulation proposed by Carver and Scheier, 1998

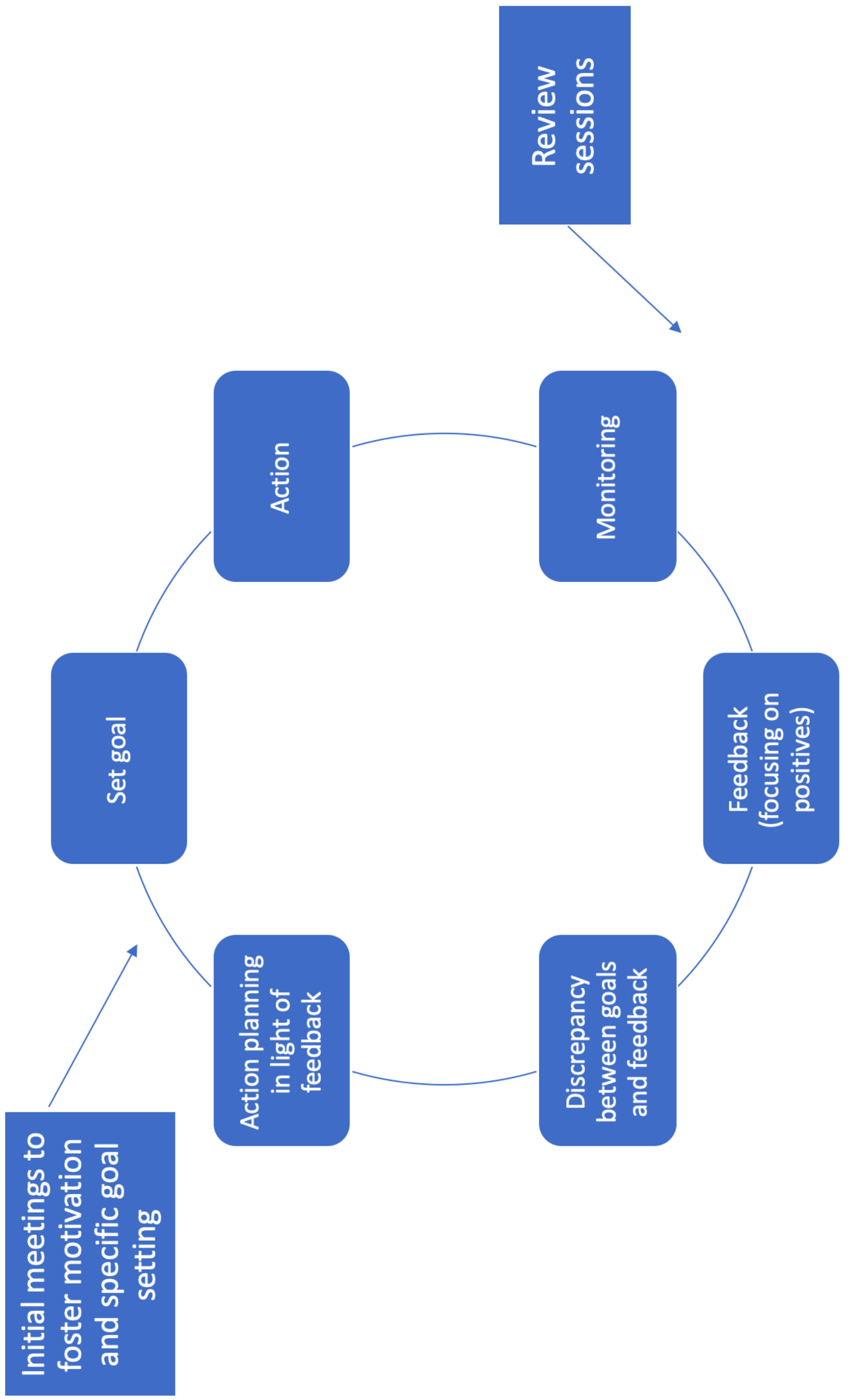

Carver CS, Scheier MF. On the self-regulation of behavior. Cambridge: Cambridge University Press, 1998.

SCENE
Enhancing social networks, improving quality of life

[INSERT TRUST LOGO]

Service User Consent Form
Randomised Control Trial of a Structured Intervention For Expanding Social Networks in Psychosis (SCENE WP5)

Please **initial** box (OR researcher to tick for remote consent)

1. I confirm that I have read and understand the information sheet dated 24/09/20 Version 3.0 for the above study. I have had the opportunity to ask the researcher questions and these questions have been answered to my satisfaction. ☐

2. I understand that my participation is voluntary and that I am free to withdraw at any time without giving any reason and without my treatment or legal rights being affected. ☐

3. I agree that if I withdraw, or am withdrawn from the study for any reason, then researchers can continue to use the information I have already given them unless I contact them before the end of July 2022 ☐

4. I understand that if I decide to stop the SCENE intervention, researchers can contact me to find out the reasons why but I am under no obligation to share this information. ☐

5. I understand that all information will be kept confidential. My personal data will only be accessed by the study team on a need to know basis. The research data will be kept for 20 years but from this, I can only be identified by a study ID code. I understand that confidentiality will need to be broken if there is a concern for risk to other people or to myself, or if criminal disclosures are made. ☐

6. I agree that members of the research team at East London NHS Foundation Trust may access my medical records to obtain basic information about me. ☐

7. I agree to take part in the above study. ☐

Please **turn over** to finish completing this form

For peer review only - http://bmjopen.bmj.com/site/about/guidelines.xhtml

Items 8-11 are **optional**. You do not have to agree to these if you do not want to. Please only **initial the boxes** for the items that you agree to.

8. I agree to be interviewed about my experiences of the SCENE intervention and for my interview to be audio recorded. I understand that the recording will be typed-up and any personal information destroyed. I agree that anonymised quotations from the interviews will be used to share the research findings. ☐

9. I agree for initial sessions of the SCENE intervention with a trained mental health professional to be audio-recorded. I understand that I will be asked for permission before each recorded session and that I am free to refuse or stop the recording at any time. ☐

10. I understand that personal information collected about me including date of birth, NHS number and postcode will be sent to the sponsor to obtain information about my service use for the purpose of this research. ☐

11. I agree to my General Practitioner (GP) being informed of my participation in this study. ☐

Name of Participant	Date	Signature<br>(OR *provided electronically by the researcher, on behalf of the participant for remote consent)*

I have explained the study to the participant and have answered the participant's questions honestly and fully

Name of Researcher	Date	Signature

Original for investigator site file, 1 copy for participant, 1 copy for medical records

For peer review only - http://bmjopen.bmj.com/site/about/guidelines.xhtml

SPIRIT

STANDARD PROTOCOL ITEMS: RECOMMENDATIONS FOR INTERVENTIONAL TRIALS

SPIRIT 2013 Checklist: Recommended items to address in a clinical trial protocol and related documents*

Section/item	Item No.	Page No.	Description
			**Administrative information**
Title	1	1	Descriptive title identifying the study design, population, interventions, and, if applicable, trial acronym
Trial registration	2a	2	Trial identifier and registry name. If not yet registered, name of intended registry
	2b	N/A (trial is ongoing)	All items from the World Health Organization Trial Registration Data Set
Protocol version	3	Date will be in published version	Date and version identifier
Funding	4	14	Sources and types of financial, material, and other support
Roles and responsibilities	5a	1,14	Names, affiliations, and roles of protocol contributors
	5b	5	Name and contact information for the trial sponsor
	5c	14	Role of study sponsor and funders, if any, in study design; collection, management, analysis, and interpretation of data; writing of the report; and the decision to submit the report for publication, including whether they will have ultimate authority over any of these activities
	5d	9	Composition, roles, and responsibilities of the coordinating centre, steering committee, endpoint adjudication committee, data management team, and other individuals or groups overseeing the trial, if applicable (see Item 21a for data monitoring committee)

Introduction

For peer review only

Background and rationale	6a	4	Description of research question and justification for undertaking the trial, including summary of relevant studies (published and unpublished) examining benefits and harms for each intervention
	6b	7	Explanation for choice of comparators
Objectives	7	4,5	Specific objectives or hypotheses
Trial design	8	5	Description of trial design including type of trial (eg, parallel group, crossover, factorial, single group), allocation ratio, and framework (eg, superiority, equivalence, noninferiority, exploratory)
			**Methods: Participants, interventions, and outcomes**
Study setting	9	5, Table 1	Description of study settings (eg, community clinic, academic hospital) and list of countries where data will be collected. Reference to where list of study sites can be obtained
Eligibility criteria	10	5	Inclusion and exclusion criteria for participants. If applicable, eligibility criteria for study centres and individuals who will perform the interventions (eg, surgeons, psychotherapists)
Interventions	11a	6,7, Box 1	Interventions for each group with sufficient detail to allow replication, including how and when they will be administered
	11b	7	Criteria for discontinuing or modifying allocated interventions for a given trial participant (eg, drug dose change in response to harms, participant request, or improving/worsening disease)
	11c	10	Strategies to improve adherence to intervention protocols, and any procedures for monitoring adherence (eg, drug tablet return, laboratory tests)
	11d	5	Relevant concomitant care and interventions that are permitted or prohibited during the trial

For peer review only - http://bmjopen.bmj.com/site/about/guidelines.xhtml

Outcomes	12	7,8	Primary, secondary, and other outcomes, including the specific measurement variable (eg, systolic blood pressure), analysis metric (eg, change from baseline, final value, time to event), method of aggregation (eg, median, proportion), and time point for each outcome. Explanation of the clinical relevance of chosen efficacy and harm outcomes is strongly recommended
Participant timeline	13	Table 2	Time schedule of enrolment, interventions (including any run-ins and washouts), assessments, and visits for participants. A schematic diagram is highly recommended (see Figure)
Sample size	14	9,10	Estimated number of participants needed to achieve study objectives and how it was determined, including clinical and statistical assumptions supporting any sample size calculations
Recruitment	15	9,10, 12-14	Strategies for achieving adequate participant enrolment to reach target sample size
			**Methods: Assignment of interventions (for controlled trials)**
Allocation:			
Sequence generation	16a	6	Method of generating the allocation sequence (eg, computer-generated random numbers), and list of any factors for stratification. To reduce predictability of a random sequence, details of any planned restriction (eg, blocking) should be provided in a separate document that is unavailable to those who enrol participants or assign interventions
Allocation concealment mechanism	16b	6	Mechanism of implementing the allocation sequence (eg, central telephone; sequentially numbered, opaque, sealed envelopes), describing any steps to conceal the sequence until interventions are assigned
Implementati on	16c	6	Who will generate the allocation sequence, who will enrol participants, and who will assign participants to interventions

For peer review only - http://bmjopen.bmj.com/site/about/guidelines.xhtml

Blinding (masking)	17a	6	Who will be blinded after assignment to interventions (eg, trial participants, care providers, outcome assessors, data analysts), and how
	17b	6	If blinded, circumstances under which unblinding is permissible, and procedure for revealing a participant's allocated intervention during the trial
			**Methods: Data collection, management, and analysis**
Data collection methods	18a	7,8	Plans for assessment and collection of outcome, baseline, and other trial data, including any related processes to promote data quality (eg, duplicate measurements, training of assessors) and a description of study instruments (eg, questionnaires, laboratory tests) along with their reliability and validity, if known. Reference to where data collection forms can be found, if not in the protocol
	18b	7,8, 12-14	Plans to promote participant retention and complete follow-up, including list of any outcome data to be collected for participants who discontinue or deviate from intervention protocols
Data management	19	7-9	Plans for data entry, coding, security, and storage, including any related processes to promote data quality (eg, double data entry; range checks for data values). Reference to where details of data management procedures can be found, if not in the protocol
Statistical methods	20a	7-9	Statistical methods for analysing primary and secondary outcomes. Reference to where other details of the statistical analysis plan can be found, if not in the protocol
	20b	8-11, 12-13	Methods for any additional analyses (eg, subgroup and adjusted analyses)
	20c	7-9	Definition of analysis population relating to protocol non-adherence (eg, as randomised analysis), and any statistical methods to handle missing data (eg, multiple imputation)
			**Methods: Monitoring**

Data monitoring	21a	9	Composition of data monitoring committee (DMC); summary of its role and reporting structure; statement of whether it is independent from the sponsor and competing interests; and reference to where further details about its charter can be found, if not in the protocol. Alternatively, an explanation of why a DMC is not needed
	21b	7-9	Description of any interim analyses and stopping guidelines, including who will have access to these interim results and make the final decision to terminate the trial
Harms	22	12	Plans for collecting, assessing, reporting, and managing solicited and spontaneously reported adverse events and other unintended effects of trial interventions or trial conduct
Auditing	23	6	Frequency and procedures for auditing trial conduct, if any, and whether the process will be independent from investigators and the sponsor
			**Ethics and dissemination**
Research ethics approval	24	12	Plans for seeking research ethics committee/institutional review board (REC/IRB) approval
Protocol amendments	25	12	Plans for communicating important protocol modifications (eg, changes to eligibility criteria, outcomes, analyses) to relevant parties (eg, investigators, REC/IRBs, trial participants, trial registries, journals, regulators)
Consent or assent	26a	12	Who will obtain informed consent or assent from potential trial participants or authorised surrogates, and how (see Item 32)
	26b	N/A	Additional consent provisions for collection and use of participant data and biological specimens in ancillary studies, if applicable
Confidentiality	27	12	How personal information about potential and enrolled participants will be collected, shared, and maintained in order to protect confidentiality before, during, and after the trial
Declaration of interests	28	14	Financial and other competing interests for principal investigators for the overall trial and each study site

For peer review only - http://bmjopen.bmj.com/site/about/guidelines.xhtml

Access to data	29	12	Statement of who will have access to the final trial dataset, and disclosure of contractual agreements that limit such access for investigators
Ancillary and post-trial care	30	N/A	Provisions, if any, for ancillary and post-trial care, and for compensation to those who suffer harm from trial participation
Dissemination policy	31a	12	Plans for investigators and sponsor to communicate trial results to participants, healthcare professionals, the public, and other relevant groups (eg, via publication, reporting in results databases, or other data sharing arrangements), including any publication restrictions
	31b	12	Authorship eligibility guidelines and any intended use of professional writers
	31c	12	Plans, if any, for granting public access to the full protocol, participant-level dataset, and statistical code
**Appendices**			
Informed consent materials	32	Appendix I	Model consent form and other related documentation given to participants and authorised surrogates
Biological specimens	33	N/A	Plans for collection, laboratory evaluation, and storage of biological specimens for genetic or molecular analysis in the current trial and for future use in ancillary studies, if applicable

*It is strongly recommended that this checklist be read in conjunction with the SPIRIT 2013 Explanation & Elaboration for important clarification on the items. Amendments to the protocol should be tracked and dated. The SPIRIT checklist is copyrighted by the SPIRIT Group under the Creative Commons “Attribution-NonCommercial-NoDerivs 3.0 Unported” license.

For peer review only
